# Inhibiting CFTR through inh-172 in primary neutrophils reveals CFTR-specific functional defects

**DOI:** 10.1038/s41598-024-82535-z

**Published:** 2024-12-28

**Authors:** Ana Lúcia Da Silva Cunha, Marfa Blanter, Janne Renders, Mieke Gouwy, Natalie Lorent, Mieke Boon, Sofie Struyf, Marianne S. Carlon

**Affiliations:** 1 Laboratory of Respiratory Diseases and Thoracic Surgery (BREATHE), Department of Chronic Diseases and Metabolism, KU Leuven, Leuven, Belgium; 2 Center for Molecular Medicine, Faculty of Medicine, KU Leuven, Leuven, Belgium; 3 Laboratory for Molecular Immunology, Department of Microbiology, Immunology and Transplantation, Rega Institute, KU Leuven, Leuven, Belgium; 4 Department of Respiratory Diseases, UZ Leuven, Leuven, Belgium; 5 Department of Pediatrics, University Hospitals Leuven, Leuven, Belgium; 6 Department of Development and Regeneration, Woman and Child, Leuven, Belgium

**Keywords:** Neutrophils, Cystic fibrosis, CFTR, Inh-172, Phagocytosis, Cell biology, Immunology, Respiratory tract diseases

## Abstract

The lungs of people with cystic fibrosis (PwCF) are characterized by recurrent bacterial infections and inflammation. Infections in cystic fibrosis (CF) are left unresolved despite excessive neutrophil infiltration. The role of CFTR in neutrophils is not fully understood. In this study, we aimed to assess which antimicrobial functions are directly impaired by loss of CFTR function in neutrophils. In order to do so, we used a specific inhibitor of CFTR ion channel activity, inh-172. CF neutrophils from PwCF harboring severe *CFTR* mutations were additionally isolated to further discern CFTR-specific functional defects. We evaluated phagocytosis, reactive oxygen species (ROS) production, neutrophil elastase (NE) and myeloperoxidase (MPO) exocytosis and bacterial killing. The inh-172 model identified decreased acidification of the phagosome, increased bacterial survival and decreased ROS production upon stimulation. In PwCF neutrophils, we observed reduced degranulation of both NE and MPO. When co-culturing neutrophils with CF sputum supernatant and airway epithelial cells, the extent of phagocytosis was reduced, underscoring the importance of recreating an inflammatory environment as seen in PwCF lungs to model immune responses in vitro. Despite low *CFTR* expression in blood neutrophils, functional defects were found in inh-172-treated and CF neutrophils. The inh-172 model disregards donor variability and allows pinpointing neutrophil functions directly impaired by dysfunctional CFTR.

**Supplementary Information:**

The online version contains supplementary material available at 10.1038/s41598-024-82535-z.

## Introduction

Cystic Fibrosis (CF) is the most common autosomal recessive disease in the Caucasian population, affecting around 1:3,500 people in the US and Western Europe^1^. It is characterized by mutations in the *CFTR* (Cystic Fibrosis Transmembrane Conductance Regulator) gene, of which over 2,000 variants have been described^2^. CFTR is a cyclic adenosine monophosphate (cAMP)-regulated adenosine triphosphate (ATP)-gated anion channel that shuttles chloride and bicarbonate across secretory epithelia, thereby regulating fluid and salt homeostasis in many different organs, including the lungs, gastro-intestinal tract and exocrine pancreas^3^.

People with CF (PwCF) acquire symptoms in many organs, although it is the lung phenotype that causes most morbidity and mortality and ultimately requires lung transplantation if left untreated. With the approval of four CFTR modulator therapies over the past twelve years, we have entered an era in which PwCF carrying one of the modulator-responsive CFTR variants are expected to live substantially longer with increased quality of life^4^. Although improvements in clinical endpoints such as lung function, body mass index, and frequency of pulmonary exacerbations were observed^5^, it is too early to understand the long-term effects on lung function and microbial colonization.

While it is well-documented that the CF airways are hyper-inflamed and chronically infected with pathogens, the causal relationship between infection and inflammation continues to be debated^6^. CFTR plays a central role in maintaining a healthy airway surface liquid that enables proper mucociliary function and protects against microbial infections. In CF, loss of bicarbonate secretion through CFTR and pendrin in airway epithelial cells causes airway surface liquid acidification, reduced antimicrobial peptide activity and impaired bacterial killing^7^. Together with impacted mucin unfolding and reduced mucociliary clearance, tumor necrosis factor-α (TNF-α) is present in the airways at early stages of the disease and is associated with increased neutrophil counts^8^ .

Neutrophils belong to the innate immune system and are recruited as first responders to fight infections. They have a multilobed nucleus and contain granules filled with antimicrobial molecules. The recruitment of neutrophils is mediated via chemoattractants, which are small proteins that are secreted by cells at the infection site to attract immune cells. Directed movement towards a high concentration of chemoattractant is called chemotaxis. Once arrived at the site of infection, neutrophils can execute four main antimicrobial functions. Their main bacterial killing mechanism is phagocytosis, i.e. neutrophils bind, engulf and degrade pathogens inside the cell. Neutrophils can recognise and bind pathogens directly using pattern-recognition receptors or indirectly using opsonin receptors^9^. The pathogen is then taken up in a phagosome, which undergoes acidification. Inside the phagosome, reactive oxygen species (ROS) are produced, which aid in the degradation of the engulfed pathogen. Secondly, neutrophils can release the content of their granules in a process called exocytosis or degranulation. Enzymes such as myeloperoxidase (MPO) and neutrophil elastase (NE) contribute to the eradication of micro-organisms. The third function of neutrophils is to release neutrophil extracellular traps (NETs), which consist of DNA strands, histones and granule proteins. NETosis (i.e. the process of NET release) is usually accompanied by the death of the neutrophil and creates toxic web-like structures that can trap and damage pathogens. Finally, neutrophils can produce cytokines and chemokines, which can influence other cells of the immune system.

CF sputum and bronchoalveolar lavage (BAL) samples show elevated levels of neutrophils and their products, including MPO and NE^10,11^. Despite their abundance, neutrophils are not able to effectively eradicate pathogens in the CF airways, suggesting that CFTR dysfunction might negatively impact neutrophil effector functions. This could be the result of either a direct effect of mutant CFTR on neutrophil function, or an indirect effect driven by an abnormal CF lung micro-environment. Evidence for both hypotheses has been reported, but so far, no consensus has been reached^12–15^. Phagocytosis has been directly linked to CFTR dysfunction, as CFTR mediates the transport of chloride into phagosomes, thus facilitating acidification^16^. Research on neutrophil effector functions can shed light on whether CFTR affects cellular functions.

In this study, we investigated whether the absence of a functional CFTR channel affects neutrophil function using two different models. In the first model, we chemically inhibited CFTR function in neutrophils from healthy donors; in the second model, we studied the function of neutrophils isolated from peripheral blood of PwCF.

## Materials & methods

A full list of reagents with catalogue numbers can be found in Table S1.

### Study participants

Peripheral blood samples were collected from 13 people with CF (PwCF) between 2023 and 2024 who were seen in the outpatient clinic, diagnosed according to the European guidelines. Nine non-CF healthy volunteers were recruited at the University Hospital of Leuven. Non-CF controls were defined as individuals over 18 years of age without any medical history of autoimmune disease or immunodeficiency, use of anti-inflammatory medication or disease symptoms. 10 mL of blood was collected in an EDTA-coated tube (BD Biosciences, Franklin Lakes, NJ, USA). All participants signed an informed consent form. The study protocol (S57236 [ML11095]) was approved by the ethical committee of KU Leuven/UZ Leuven.

### Neutrophil purification

Neutrophils were purified using the EasySep Direct Human Neutrophil Isolation kit (StemCell Technologies, Vancouver, BC, Canada) as previously described^17^. Briefly, blood was divided over 15-mL tubes and supplemented with Isolation Cocktail (50 µL per 1 mL of blood) and Rapidspheres™ (50 µL per 1 mL blood), followed by a 5-min incubation at room temperature (RT). During this step, non-neutrophilic cells were labeled with antibodies against lineage markers connected to magnetic beads. Subsequently, the blood was supplemented with Dulbecco’s phosphate-buffered saline (D-PBS; VWR, Radnor, PA, USA) to a volume of 10–12 mL, whereupon the tubes were placed for 10 min in an EasyEights magnet (StemCell Technologies). During this step, labeled (i.e. non-neutrophilic) cells were bound to the magnet. The unbound fraction was then transferred to a clean tube and incubated for 5 min with Rapidspheres™ (50 µL per 1 mL of initial blood volume). The tube was placed in the magnet and incubated for another 5 min, whereupon the unbound fraction was transferred to a clean tube and the magnet incubation step was repeated. Lastly, the enriched cell suspension was collected and spun down (8 min, 177 g, RT). The pellet was resuspended in D-PBS. To determine the cell concentration, a small aliquot of the cell suspension was stained with Türk’s solution (Sigma-Aldrich, Saint Louis, MO, USA) and counted in a Bürker chamber.

### RNA extraction and cDNA conversion

RNA from neutrophils was extracted using the RNeasy^®^ Micro Kit (Qiagen) according to the manufacturer’s protocol. 2 × 10^6^ neutrophils were used per sample. Briefly, cells were washed with PBS, lysed and centrifuged in a spin column to remove genomic DNA. The flow-through was saved and supplemented with 70% ethanol in a 1:1 ratio, whereupon the mixture was transferred to a spin column. The sample was centrifuged at maximum speed. The sample was treated with different solutions supplied with the kit, and eluted in RNAse-free water. The concentration of RNA was measured using a nanophotometer (Nanophotometer NP80, Implen). High-Capacity cDNA Reverse Transcription Kit (Applied Biosystems™) was used to generate cDNA according to the kit formulation. Briefly, a 1:1 mixture of RNA and a 2x mix containing RT Buffer, dNTP Mix, Random Primers, Reverse Transcriptase, and nuclease-free water was made and placed in the thermocycler (Biometra^R^ Tone) for 10 min at 25 °C, followed by 2 h at 37 °C and, lastly, 10 min at 85 °C.

### CFTR expression detection by nested PCR

To detect the expression of CFTR RNA, two polymerase chain reactions (PCRs) were performed. In the first PCR, a Taq Polymerase (ThermoFisher, Waltham, MA, USA) mix was composed according to the manufacturer’s instructions with initial cDNA concentration set at 4,5 µg/mL. Forward (Fw) primer 1 and reverse (Rev) primer 2, listed in Table 1 were used at 5 µM. The PCR was performed at an annealing temperature (T_a_) of 54 °C and an elongation time of 60 s for a total of 40 cycles. The resulting amplicon length was 777 base pairs (bp). The amplicon was loaded on a 2% agarose gel in TAE (Tris-Acetate EDTA) buffer and run for 45 min at 145 V. Agarose gel images were captured using Molecular Imager GelDoc XR^+^ (Bio-Rad), with automatic setting of brightness and contrast. No additional adjustments were made to the acquired images. After identification of a band on the positive control sample, a PCR cleanup (Genelute PCR Clean-up Kit, Sigma-Aldrich) was performed to remove primer dimers. 1 µL of the resulting PCR product was used for the second PCR using the same mix and primer 3 (Fw) and primer 4 (Rev) at 5 µM (Table 1). For this PCR, the T_a_ was set at 54 °C and the number of cycles at 35. The resulting 275-bp PCR amplicon was loaded on a 2% agarose gel in TAE buffer and run for 45 min at 145 V. Agarose gel images were captured as described above. After PCR cleanup, the samples were subjected to Sanger sequencing to verify the exact sequence of the resulting CFTR amplicon.

**Table 1:** List of primers used for nested PCR to detect CFTR cDNA.

Primer 1 (Forward)	GGCACATTTCGTGTGGATCGCTCC
Primer 2 (Reverse)	ACCGCCAACAACTGTCCTCTT
Primer 3 (Forward)	GCATACTGCTGGGAAGAAGC
Primer 4 (Reverse)	AGAGAGTCATACCATGTTTGTACAGC

### Immunofluorescence for CFTR detection in neutrophils

To detect the presence of the CFTR protein, an immunofluorescence staining was performed. A Nunc™ Lab-Tek™ Chambered Coverglass (Thermo Scientific™) was coated with poly-L-lysine (100 µg/mL; Sigma-Aldrich) for 1 h at RT, whereupon it was washed twice with sterile PBS and air-dried. Neutrophils (0.3 × 10^6^ cells/well) were suspended in RPMI medium and added to the coated chamber; the chamber was then incubated for 30 min at 37 °C. Subsequently, the medium was removed and the cells were fixed with 4% paraformaldehyde (Alfa Aesar, Heysham, United Kingdom) for 15 min at RT. Following the fixation, the cells were washed twice with HBSS buffer (ThermoFisher Scientific) and permeabilized with 0.1% Triton-X (Merck) for 15 min at RT. The cells were washed twice with HBSS and incubated for 30 min at RT in blocking agent (PBS + 10% fetal bovine serum [FBS; Sigma-Aldrich] + 1% bovine serum albumin [BSA; Sigma-Aldrich]). After the blocking step, the cells were incubated with mouse monoclonal anti-human CFTR antibodies (diluted 1:250, CFTR antibody mix 570, 596 & 660 from CFFT labs, www.CFF.org) for 1 h at RT. Subsequently, the antibody was removed and the cells were washed three times with HBSS buffer. A secondary antibody (Donkey Anti-Mouse Alexa Fluor 488, diluted 1:500, Invitrogen) was added and incubated for 1 h at RT. The cells were then washed three times with HBSS buffer and kept at 4 °C in the dark until imaging. Image acquisition was performed with a Leica Dmi8 with 63x/1.2 lens. Zoomed images were obtained in ImageJ by drawing a rectangle of approximately 50 μm x 50 μm, using the command *Crop* and *Set scale.*

### Phagocytosis assay

A 96-well black clear-bottom plate (Greiner Bio-One, Kremsmünster, Austria) was coated with poly-L-lysine (100 µg/mL; Sigma-Aldrich) for 1 h at 37 °C, whereupon it was washed twice with sterile water and air-dried. Neutrophils were suspended in phagocytosis buffer (Live Cell Imaging Solution [Gibco, Billings, MT, USA] + 20 mM HEPES [Gibco]) at a density of 0.5 × 10^6^ cells per mL. Cells were added to the coated plate (100 µL/well) and incubated with calcein AM (1 µM; Invitrogen, Waltham, MA, USA) and either inhibitor-172 (inh-172, 50 µM, Selleckchem) or DMSO (0.5%, Sigma-Aldrich) for 30 min at 37 °C, 5% CO_2_. Subsequently, phagocytosis buffer alone or N-formyl-methionyl-leucyl-phenylalanine (fMLP; Sigma-Aldrich, final concentration 10^− 7^ M) diluted in phagocytosis buffer were added to the cells, followed by a 10-min incubation at 37 °C. FlashRed beads (Bangs Laboratories, Fishers, IN, USA; 0.5 × 10^6^ beads/well) were opsonized for 1 h at 37 °C in PBS with human serum prior to incubation with neutrophils. Finally, either pHrodo^TM^-labelled *S. aureus* bioparticles (Invitrogen; final concentration 62.5 µg/mL) or FlashRed beads were added to the plate and incubated for 2–3 h at 37 °C, whereupon microscopy pictures (phase-contrast, red fluorescence and green fluorescence) were taken at a 20x magnification using the Incucyte S3 live cell imaging system (Sartorius, Göttingen, Germany). pHrodo™ is a pH-sensitive dye that emits red fluorescence upon acidification, which happens during maturation of the phagosome. Inh-172 and DMSO were added at each step to ensure a constant concentration of 50 µM or 0.5%, respectively, throughout the experiment.

Image analysis was performed using the integrated Incucyte software for the PwCF samples. Pre-processing of the fluorescent signals was performed. In the green channel (live cell – Calcein), the processing was accomplished by applying watershed transformation and filtering out cell aggregates. Additionally, in the red channel (signal of pHrodo^TM^-labelled *S. aureus* bioparticles), removal of large aggregates of particles or low background fluorescence signal was done. A cell was considered positive for phagocytosis if an overlap of red and green was observed.

Image analysis for the inh-172 experiment was performed using the raw images provided by Incucyte software in macro language for ImageJ. Briefly, the green channel (live cell – Calcein) images were converted into binary images using an automatic threshold. Watershed transformation was applied. Cell aggregates and debris were removed. A summary table of the results is provided (Suppl. File 1). With the images from the green and the red channel (signal of pHrodo^TM^-labelled *S. aureus* bioparticles ), a composite was created (Suppl. File 2). The composite was saved and split into blue, green and red channels. Values of intensity above 125 (scale 0-255 for 8-bit) on both red and green channels (resulting in yellow - phagocytosed bacteria) were analysed and a new binary image was created (Suppl. File 3). An additional overlay with the binary neutrophil image (green channel, calcein positive, live neutrophils) was done in order to discriminate overlap with live neutrophils from overlap with debris (Suppl. File 4). A cell was considered positive for phagocytosis if an overlap of the binary images for yellow (phagocytosed particle) and green (viable neutrophil) was observed.

### Actin polymerization

Neutrophils (1.5 × 10^6^ cells/mL) were suspended in colorless RPMI medium containing 1 mg/mL human serum albumin (Belgian Red Cross) and either Inh-172 (50 µM) or DMSO (0.5%), followed by a 30-min incubation at 37 °C. Subsequently, 70 µL of the cells were transferred for 30 s to a pre-warmed U-bottom 96-well plate containing CXCL8 (10 ng/mL), fMLP (10^− 7^ M), C5a (R&D; 10 ng/mL) or leukotriene B_4_ (LTB_4_; Cayman chemicals, Ann Arbor, MI, USA; 5 ng/mL). Following the 30 s-incubation, the cells were fixed and permeabilized with BD CytoFix/CytoPerm buffer (BD Biosciences, Franklin Lakes, NJ, USA) for 20 min at 4 °C. The cells were then resuspended in BD Perm/Wash buffer (BD Biosciences) containing 2 U/mL AF555-Phalloidin (Invitrogen) and incubated for 20 min at 4 °C. Subsequently, the cells were washed twice with BD Perm/Wash Buffer and resuspended in PBS containing 2% v/v FCS and 2 mM EDTA. The cells were kept at 4 °C until further analysis. Quantification of the fluorescence was performed by flow cytometry using a BD LSRFortessa™ X-20 (BD Biosciences) equipped with DIVA software (BD Biosciences). Downstream analysis was performed using FlowJo software (BD Biosciences, v.10.8.1).

### Total reactive oxygen species (ROS) chemiluminescence assay

To measure the capacity of neutrophils to produce reactive oxygen species, a luminol-based chemiluminescence assay was employed. Neutrophils were suspended in RPMI-1640 medium without phenol red (Gibco) at a density of 1.5 × 10^6^ cells/ml in the presence of either Inh-172 (50 µM) or DMSO (0.5%) and incubated for 30 min at 37 °C, 5% CO2. Subsequently, the cells were supplemented with either medium alone or TNF-α (25 ng/ml; Peprotech) diluted in medium, and incubated for 10 min at 37 °C. After 10 min, the cells were plated in a white, clear-bottom 96-well microplate (Perkin Elmer, Waltham, MA, USA) together with luminol (Sigma-Aldrich; final concentration 5 mM) and one of the following inducers: buffer, phorbol myristate acetate (PMA; Sigma-Aldrich; 150 ng/ml), N-Formylmethionine-leucyl-phenylalanine (fMLP, 10^− 7^ M); peptidoglycan (PGN) from *S. aureus* (Sigma-Aldrich, 10 µg/ml) or lipopolysaccharide (LPS, 10 µg/ml) from *E. coli* (Sigma-Aldrich; 10 µg/ml). The luminescence emitted through luminol oxidation was measured every 2 min for 2 h at 37 °C using a Clariostar monochromator microplate reader (BMG Labtech, Orthenberg, Germany). Peak luminescence was plotted for each condition. Background luminescence was determined by including a condition with PMA without luminol; the mean of these measurements was subtracted from all sample values. Inh-172 and DMSO were added at each step to ensure a constant concentration of 50 µM or 0.5%, respectively, throughout the experiment.

### Degranulation assay

Neutrophils (1.5 × 10^6^ cells/mL) were suspended in RPMI medium (Gibco) with either Inh-172 (50 µM) or DMSO (0.5%) and incubated for 30 min at 37 °C. Subsequently, the cells were supplemented with either medium, fMLP (final concentration 10^− 6^ M) or LPS (final concentration 5 µg/mL and 10 µg/mL for inh-172 and patient experiments, respectively). The cells were subsequently incubated for 2 h at 37 °C. Thereafter, the supernatant was collected and stored at -20 °C until further analysis. Levels of myeloperoxidase (MPO) and neutrophil elastase (NE) were measured with DuoSet ELISAs (R&D Systems), according to the manufacturer’s instructions.

### Bacterial killing assay

*Escherichia coli* bacteria (strain DH5α) were inoculated in lysogeny broth (LB) medium and grown overnight at 37° C while shaking. Following the overnight incubation, the optical density (OD) value was measured at 600 nm using a spectrophotometer to verify exponential growth (OD = 1). The number of colony-forming units (CFU) was calculated based on an* E.coli*-specific formula established through a calibration curve; OD 1 = 3 × 10^8^ CFU/mL. While neutrophils were isolated from whole blood of healthy donors, a 12-well plate (TPP, Trasadingen, Switzerland) was coated with poly-L-lysine (100 µg/mL; Sigma-Aldrich) for 1 h, whereupon it was washed twice with sterile water and air-dried. After purification, neutrophils were resuspended in HBSS and treated with DMSO (0.5%), as solvent control, or Inh-172 (50 µM). Neutrophils were added to the plate at a concentration of 1 × 10^6^ cells per well and incubated for 30 min at 37 °C. A predilution of bacteria was prepared in HBSS buffer for a multiplicity of infection (MOI) of 3. Bacteria were opsonized with 10% human serum for 30 min while shaking at 37 °C. Afterwards, bacteria were spun down at 10,000 g, resuspended in HBSS, and added to the plate containing neutrophils. HBSS buffer was added to each well until a total volume of 1 ml was obtained. To assess normal bacterial growth, a well without neutrophils was included. To speed up the contact with the neutrophils, the plate was centrifuged (5 min, 300 g) and thereafter placed in the incubator at 37 °C and 5% CO_2_ for 2 h to allow bacterial killing. After incubation, the supernatant was collected, containing the extracellular, living bacteria. The pellet was resuspended in 2% saponin (Sigma-Aldrich) in ultrapure water for 30 min at RT, in order to lyse the neutrophils and collect the intracellular bacteria. Both the intracellular and extracellular fractions were diluted in ultrapure water according to the following dilution series; 10^− 1^, 10^− 2^ and 10^− 3^. To assess bacterial growth, the dilutions were plated on MacConkey agar plates (Sigma-Aldrich). Of each dilution, 20 µL was plated in triplicate on an agar plate. The plates were shortly air-dried and incubated overnight at 37 °C. The next day, bacterial colonies were counted and the number of CFU was calculated.

### CF sputum supernatant

The CF sputum was mixed 1:1 with a sterile solution of 0.1% (w/v) dithiothreitol (DTT). The solution was vortexed for 5–10 min, transferred to a clean tube, and shaken for an additional 15–20 min. Then, the samples were centrifuged for 10 min at 16,000 g and the supernatant was stored at -80 °C. A pool of CF sputum supernatants was formed with 5 different samples, which were aliquoted and stored at -80 °C until use.

### 16HBE14o- airway epithelial cell culture

The Human Bronchial Epithelial Cell Line 16HBE14o- (Merck) was grown in MEM medium (Gibco) supplemented with 10% FBS and 50 ng/mL of gentamicin. The culture plates were coated with 0.01% BSA (Gibco), 0.03 mg/mL of PureCol (Merck) and 10 µg/mL of fibronectin from human plasma (Thermo Fisher) in LHC-8 basal medium (Gibco) for 2 h at 37 °C; the coating solution was removed and the plates were used immediately or stored at 4 °C. The medium was changed three times a week until approximately 80% confluency was reached. For cell passaging, the medium was aspirated and the cells were washed once with D-PBS. Then, cultures were incubated with 1-2 mL of TrypLE^TM^Express (Thermo Fisher) for 7 min at 37 °C. Growth medium was added up to 10 mL to neutralize the enzyme. The cell suspension was centrifuged for 3.5 min at 0.1 g. The supernatant was aspirated and the cells were resuspended in growth medium. Cell counting was performed using Trypan Blue (Thermo Fisher).

### Phagocytosis assay after co-culture with 16HBE14o- and/or CF sputum

96-well plates (VWR) were coated (50 µL/well) as described above and 50,000 epithelial cells were added per well. After 1 day in culture, the cells formed a monolayer. 2–3 h before adding neutrophils, the medium was replaced by growth medium containing 20% (v/v) of either the pool of CF sputum supernatants or 0.1% (v/v) DTT or D-PBS with either inh-172 at a final concentration of 50 µM or 0.5% DMSO (v/v). In all conditions, GM-CSF (granulocyte-macrophage colony-stimulating factor) was present at a final concentration of 50 ng/mL. Neutrophils at a density of 5 × 10^6^ cells/mL were incubated with either inh-172 (at a final concentration of 50 µM) or DMSO (0.5%) for 30 min at 37 °C. 50,000 cells were added on top of each well. The control condition did not include the epithelial monolayer. After 2 h of co-culture, neutrophils were aspirated and centrifuged at 0.1 *g* for 8 min at RT. A phagocytosis assay was performed as described above, with the addition of GM-CSF at a final concentration of 50 ng/mL.

### Viability assessment

A 96-well black clear-bottom plate (Greiner Bio-One) was coated (50 μL/well) as described above and 50,000 epithelial cells were added per well. After 1 day in culture, the cells formed a monolayer. 2–3 h before adding neutrophils, the medium was replaced by growth medium containing 20% (v/v) of either the pool of CF sputum supernatants or a 0.1% (v/v) DTT or D-PBS or PMA (as positive control for NETosis), at a concentration of 100 µg/mL with either inh-172 at a final concentration of 50 µM or 0.5% DMSO (v/v). Sytox Green (Invitrogen) was added to all conditions at a final concentration of 50 nM. In all conditions, GM-CSF was present at a final concentration of 50 ng/mL. The control condition did not include neutrophils but only epithelial cells exposed to the different stimuli to determine the background. Neutrophils at a density of 5 × 10^6^ cells/mL were incubated with either inh-172 (at a final concentration of 50 µM) or DMSO (0.5%) for 30 min at 37 °C. 50,000 cells were added on top of each well. Microscopy pictures (phase-contrast and green fluorescence) were taken at a 20x magnification using the Incucyte S3 live cell imaging system (Sartorius, Göttingen, Germany) for 2 h.

Image analysis was performed using the raw images provided by Incucyte software in macro language for ImageJ. Briefly, a subtraction of background fluorescence was performed on the green channel images (staining of free nucleic acid by Sytox Green). The images were converted to 8-bit. A threshold based on mean grey intensity was applied. Images were converted into binary images. A summary table of the results is saved containing the total Sytox Green positive area. For representation, a normalization to the background signal in the 16HBE14o- only condition was performed (Suppl. File 5).

### Statistical analysis

All differences between control and inh-172 or PwCF groups were statistically analyzed by nonparametric, non-paired Kolmogorov-Smirnov test for PwCF data and nonparametric, paired Wilcoxon matched-pairs signed rank test for the inh-172 data using GraphPad Prism version 10.0.2. P-values below 0.05 were considered statistically significant and represented with asterisks: * for *p* ≤ 0.05, ** for *p* ≤ 0.01 and *** for *p* ≤ 0.001. P-values above 0.05 were not represented. A ROUT (Robust Regression and Outlier Removal) was performed to identify outliers in the inh-172 model. If an outlier value was identified in more than one stimulus in a single experiment, the full experiment was omitted. Otherwise, the single outlier was removed. For experiments with a small number of biological repeats, a Shapiro-Wilk test was used to assess the normality of the data to allow the use of paired t tests as replacement for paired Wilcoxon matched-pairs signed rank test. The specific test used is detailed in the legend of each figure.

## Results

### Primary neutrophils express CFTR

**Fig. 1:**
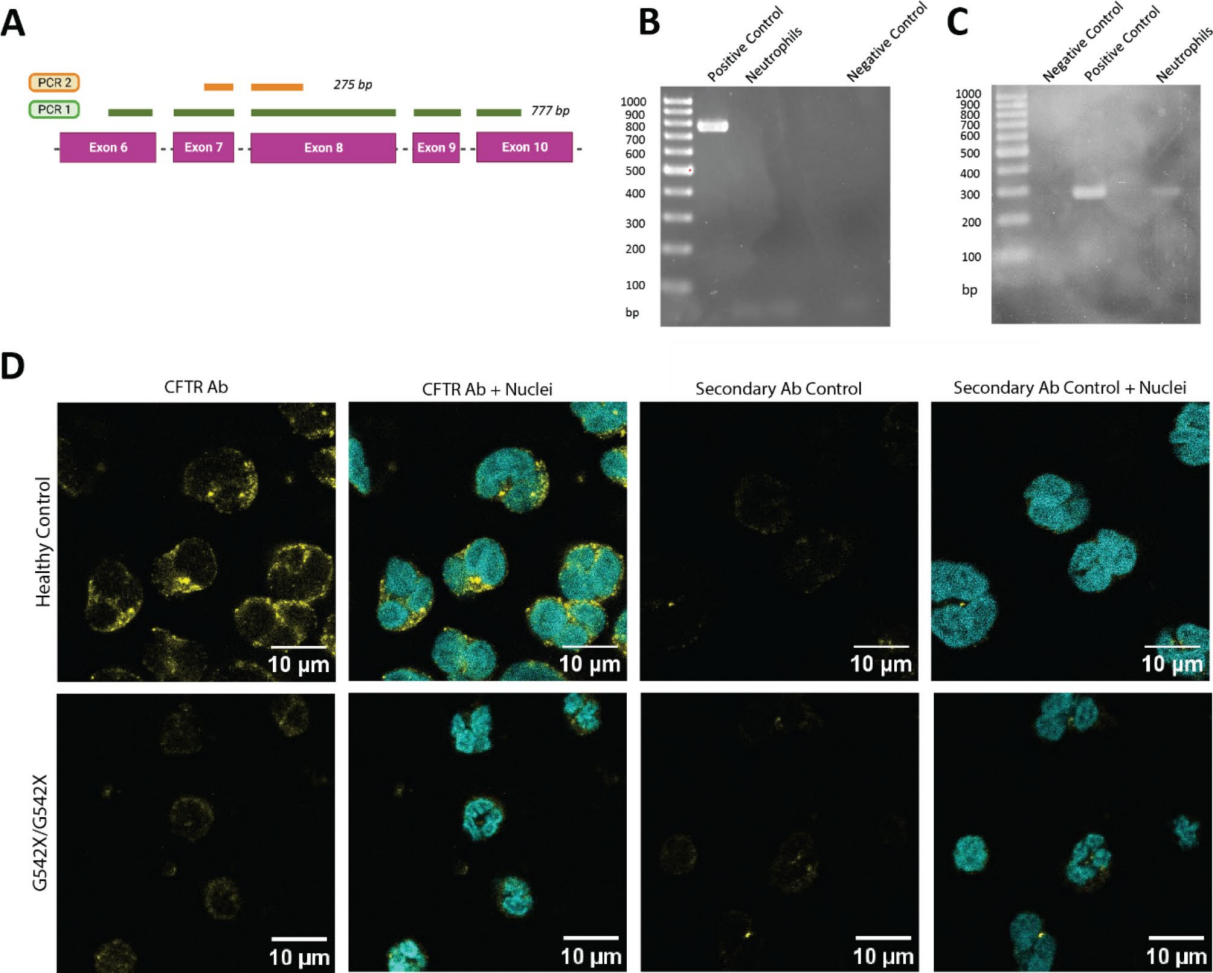
Detection of CFTR expression in primary non-CF neutrophils. **(A)** Schematic view of the two PCR products generated by nested PCR to detect *CFTR* mRNA expression on generated *CFTR* cDNA. **(B)***CFTR* cDNA from neutrophils of a representative healthy donor, HEK293T-overexpressing CFTR (*positive control*), and negative control (*water*) was amplified using Taq Polymerase in the first round of PCR (PCR1). **(C)** A second PCR (PCR2) was performed on the purified PCR1 amplicon. The obtained PCR2 amplicon was subjected to Sanger sequencing to confirm the correct CFTR sequence (*Suppl. Figure 1*). **(D)** CFTR staining of primary neutrophils. Immunofluorescence images show primary neutrophils from a healthy donor (top row) and a PwCF homozygous for the G542X mutation (bottom row). Samples were stained for CFTR (yellow) and counterstained with the nuclear stain DAPI (cyan) in the first two columns. The last two columns show immunofluorescence from the secondary antibody only, serving as a control in the third column, and combined with DAPI in the fourth column. Images from six additional healthy donors are provided in *Supp.* Figure 2. The scale bar represents 10 μm.

To confirm the expression of CFTR in primary neutrophils, we performed a nested PCR on mRNA extracted from healthy neutrophils (Fig. 1A-C). As a positive control, we used either a cell line that endogenously expresses high levels of CFTR (Caco2) or an engineered cell line with overexpression of *CFTR* cDNA^18^. The first PCR showed amplification of the positive control sample (Fig. 1B, *Suppl. Figure 1A*,* C*,* E*), while no band was seen for the neutrophil samples. In the second PCR, a band was present for both samples, indicating that *CFTR* mRNA expression in neutrophils at rest (i.e. without stimulation) is detectable, though low (Fig. 1C, *Suppl. Figure 1B*,* D*,* F*). Sanger sequencing confirmed the correct *CFTR* sequence of the amplicon *(Suppl. File 6*). Next, we used immunocytochemistry to investigate CFTR expression at the protein level. Varying levels of CFTR signal were detected throughout the cytoplasm in seven healthy donors and absent in the CF sample with two class I mutations (G542X/G542X), implying absence of CFTR protein (Fig. 1D, *Supp.* Figure 2).

### Inhibition of CFTR function in neutrophils reduces phagosome acidification

To study intrinsic CFTR-related defects in peripheral blood neutrophils, we opted for two CF models: PwCF peripheral blood neutrophils on the one hand, and chemically inhibited CFTR on the other hand. We reasoned that the application of the small molecule CFTR inhibitor, inh-172, widely used in different cell types including neutrophils^19–21^, would reduce donor-to-donor variability, as the control condition would have the same donor sample as the CFTR-inhibited condition.

**Table 2:** PwCF and healthy control characteristics for functional assays.

	People with CF	Healthy Controls
N	9	9
Sex (female/male)	3/6	6/3
Median Age (years)	27	29
Age Range (years)	20–37	23–42

**Table 3:** Characteristics of included PwCF.

Symbol	CFTR variant 1	CFTR variant 2	FEV_1_ (% predicted)	Sweat chloride (mmol/L)	Exocrine pancreatic insufficiency	Infection status
$\blacksquare$	W1310X	2183AA > G	36%	**123**	Yes	*Pseudomonas aeruginosa*
$\blacktriangle$	F508del	F508del	86%	**92**	Yes	*Pseudomonas aeruginosa*
♦	F508del	C276X	77%	**112**	Yes	*Achromobacter xylosoxidans*
$\otimes$	F508del	N1303K	116	**96**	Yes	*Staphylococcus aureus*
●	3120 + 1G > A	1677delTA	88%	**116**	Yes	*Staphylococcus aureus*,* Stenotrophomnas maltophilia*,* Aspergillus fumigatus *c*omplex*
$\circ$	F508del	F508del	107%	**110**	Yes	*Staphylococcus aureus*
$\triangle$	N1303K	4218INST	91%	**114**	Yes	*Achromobacter xylosoxidans*
$\ast$	N1303K	N1303K	101%	**99**	Yes	*Pseudomonas aeruginosa (mucoid)*, *Staphylococcus aureus*,* Aspergillus fumigatus complex*
×	F508del	F508del	79%	**111**	Yes	*Staphylococcus aureus*
	G542X	G542X	90%	**117**	Yes	*Staphylococcus aureus*,* Pseudomonas aeruginosa*

Symbols are used to identify individual patients in the graphs. Exocrine pancreatic insufficiency is annotated as present (‘yes’). Patient G542X/G542X was included only as a control for the immunostaining of CFTR. Abbreviations: FEV1: forced expiratory volume in one second.

**Fig. 2:**
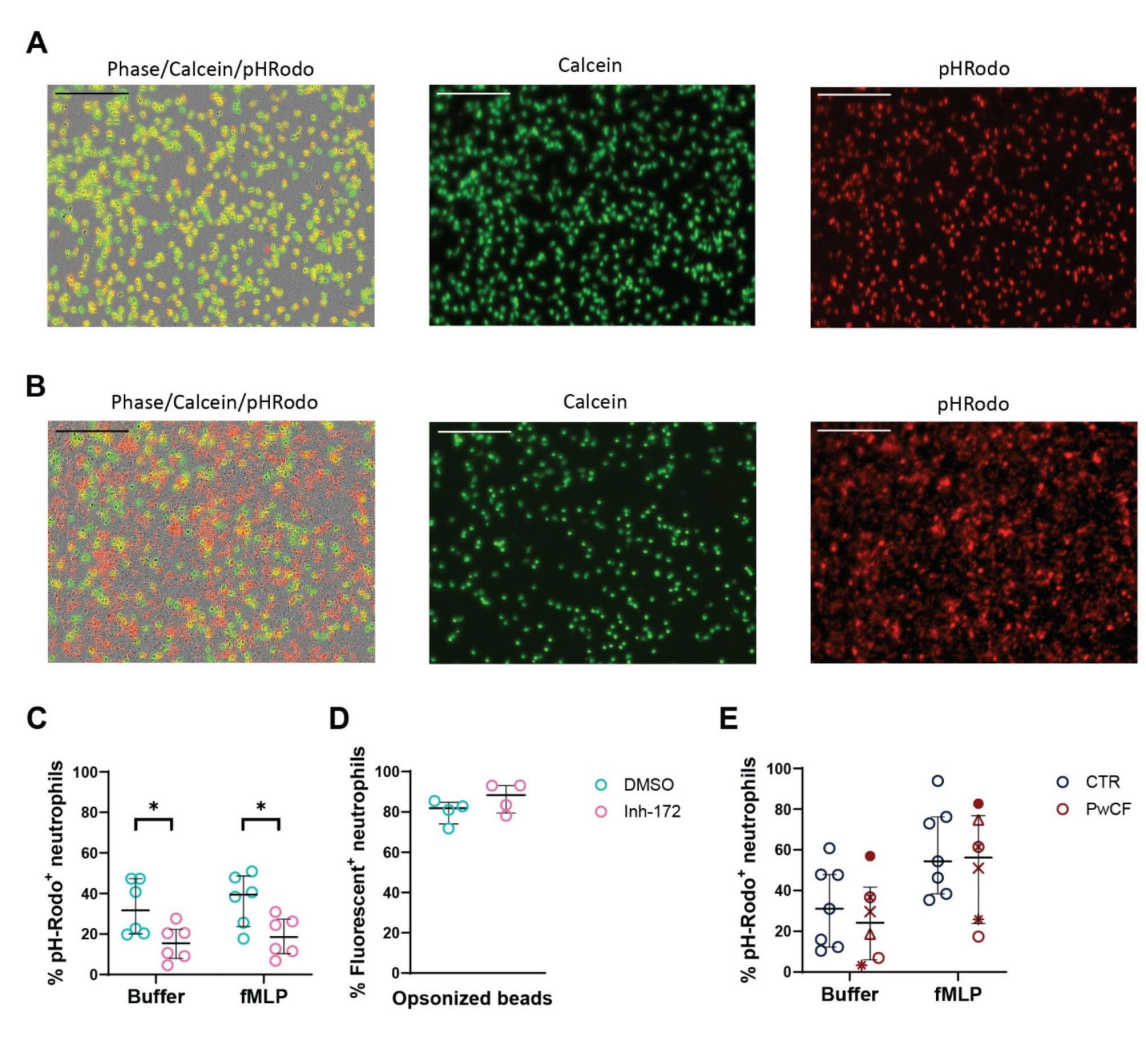
Effect of CFTR dysfunction on phagocytosis by neutrophils. Representative microscopic picture of healthy donor neutrophils treated with **(A)** vehicle or **(B)** inh-172 and incubated for 2 h with *Staphylococcus aureus* bioparticles. Green color indicates calcein staining; red color indicates pHrodo labelling. The size of the scalebar is 200 μm. **(C)** Healthy donor neutrophils were pre-incubated for 30 min with inh-172 (CFTR-inhibitor) or DMSO (vehicle) and labelled with calcein, whereupon the cells were primed for 10 min with buffer or fMLP and exposed to pHrodo^TM^-labelled *S. aureus* bioparticles. The cells were subsequently microscopically imaged for 2–3 h. Each data point represents the median of the percentage of pHrodo-positive cells. **(D)** Healthy donor neutrophils were pre-incubated for 30 min with inh-172 or vehicle (DMSO), followed by exposure to serum-opsonized Flash Red fluorescent beads. Each data point is the median percentage of neutrophils containing at least one bead after 2–3 h of incubation. **(E)** Neutrophils from healthy donors (CTR) or PwCF were labelled with calcein, whereupon the cells were primed for 10 min with buffer or fMLP and exposed to *S. aureus* bioparticles. The cells were subsequently microscopically imaged for 2–3 h. Each data point represents the median of the percentage of pHrodo-positive cells. The legend for each symbol of PwCF is displayed in Table 3. *Supp.* Figure 3 shows the subdivision per mutation class. Graphs represent the data as median ± interquartile range. Statistical differences were determined using the Wilcoxon matched-pairs signed rank test for inh-172 data and the Kolmogorov-Smirnov test for PwCF data.

First, we investigated the role of CFTR in the process of phagocytosis (i.e. engulfment and subsequent acidification of the phagosome) by adding pHrodo^TM^-labelled *S. aureus* bioparticles to CFTR-inhibited and control neutrophils (Fig. 2). Representative images of healthy donor neutrophils treated with vehicle or inh-172 and incubated for 2 h with *S. aureus* bioparticles are shown in Fig. 2A and B (calcein staining of live neutrophils shown in green; pHrodo-labelled *S. aureus* bioparticles shown in red). Inhibition of CFTR resulted in a lower number of neutrophils with overlapping calcein and pHrodo signals in basal and fMLP-stimulated conditions as compared to the condition without inhibitor (Fig. 2C), indicating impaired phagocytosis. To distinguish between reduced particle uptake and defective phagosome acidification, we repeated the experiment with serum-opsonized Flash Red fluorescent beads, which do not depend on the pH of their environment for their emitted fluorescence. We observed no reduced uptake of Flash Red beads upon CFTR inhibition (Fig. 2D). Taken together, these results indicate that inhibition of CFTR leads to diminished phagosome acidification but does not impair physical particle uptake.

Subsequently, we tested the phagocytosis capacity of peripheral blood neutrophils from PwCF (Fig. 2E). Tables 2 and 3 describe the characteristics of PwCF and respective controls. Selection of PwCF was done based on the absence of CFTR modulator intake to avoid a potential confounding effect on CFTR function and related to that, neutrophil effector function. The nine included PwCF for the functional assays were selected as presenting with severe CF based on their high sweat chloride levels (> 60 mmol/L), exocrine pancreas insufficiency and both *CFTR* variants belonging to mutation classes I or II. Overall, neutrophils from PwCF showed no significant difference in the degree of phagocytosis compared to healthy controls, either at baseline or primed with fMLP (Fig. 2E; *Supp.* Figure 3). However, the variability observed in both groups was high.

### Acute CFTR inhibition in neutrophils has no effect on actin polymerization

**Fig. 3:**
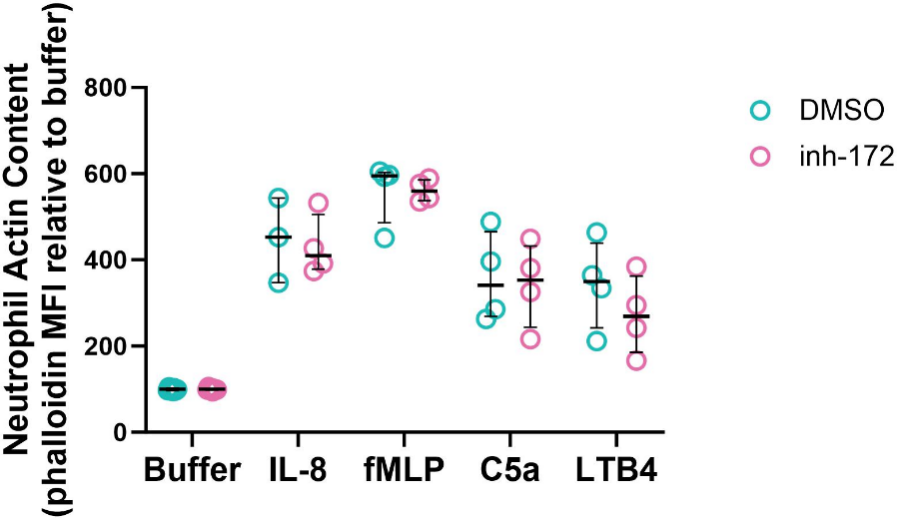
Effect of CFTR inhibition on actin polymerization by neutrophils. Neutrophils from healthy donors were incubated in the presence or absence of inh-172 (50 µM, 30 min), whereupon the cells were exposed for 30 s to a chemoattractant. The cells were then fixed, permeabilized and stained with fluorescently labelled phalloidin, which binds to polymerized actin. Median fluorescence intensity (MFI) of phalloidin staining after exposure to buffer, CXCL8 (IL-8), fMLP, C5a or LTB4. Statistical differences between treatment groups were determined using Wilcoxon matched-pairs signed rank test.

During the first steps of chemotaxis, neutrophils undergo a process called actin polymerization, in which filamentous actin is formed on one side of the cell, establishing cell polarity, a process that occurs within less than one minute^22^. To determine whether CFTR function is necessary for actin polymerization to occur, we treated healthy donor neutrophils with CFTR inhibitor inh-172 and subsequently exposed them shortly to either buffer or a chemoattractant. We measured an increased filamentous actin content in cells exposed to IL-8, fMLP, C5a and LTB4, but we observed no difference in neutrophils that were treated with inh-172, as compared to those that were treated with vehicle (Fig. 3).

### ROS production by CFTR-inhibited neutrophils is reduced

**Fig. 4:**
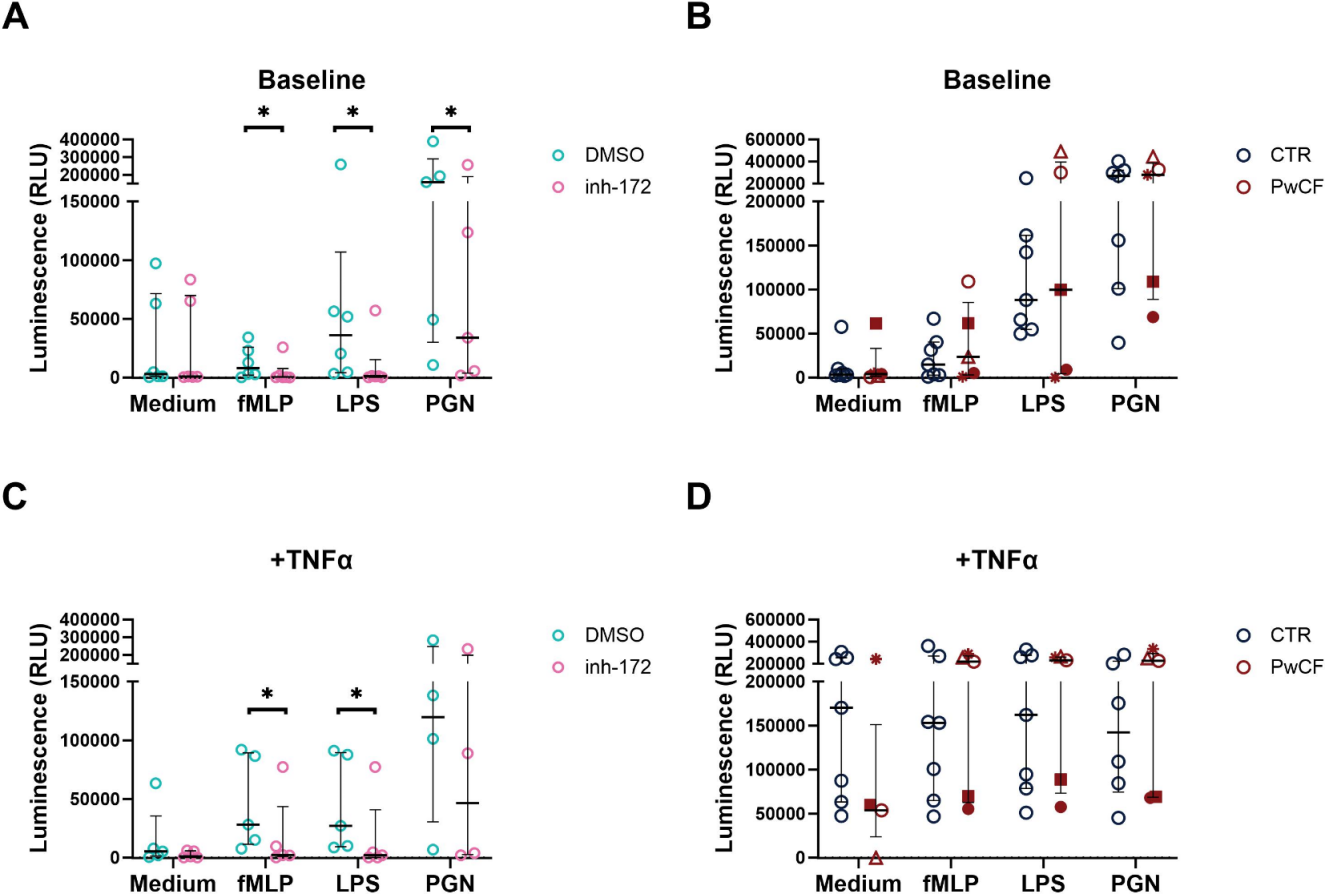
ROS production by CFTR-inhibited and PwCF neutrophils. Reactive oxygen species (ROS) production, quantified using a luminol-based chemiluminescence assay, by neutrophils from healthy donors (CTR), with or without CFTR-specific inhibition (‘inh-172’ or ‘DMSO’, respectively) or from people with cystic fibrosis (PwCF) was induced with medium, N-formyl-methionyl-phenylalanine (fMLP), lipopolysaccharide (LPS) or peptidoglycan (PGN). Tumor necrosis factor-alpha (TNF-α) was added as a priming agent to enhance neutrophil function. Peak luminescence is shown for medium, fMLP, LPS, PGN. Baseline ROS production without TNF-α for the **(A)** inh-172 model and **(B)** PwCF compared to CTR. ROS production with TNF-α priming for the (**C**) inh-172 model and **(D)** PwCF compared to CTR. Statistical differences were determined using Wilcoxon matched-pairs signed rank test for inh-172 data and Kolmogorov-Smirnov test for PwCF data. *Supp.* Figure 4 shows the subdivision per mutation class. RLU: relative light units.

Reactive oxygen species (ROS) can be produced intracellularly in the phagosome following phagocytosis of pathogens or released into the extracellular environment upon neutrophil activation^9^. Whether CFTR dysfunction affects ROS production in CF neutrophils is to date poorly understood. To gain insights in potential differences in ROS production, we quantified ROS levels by chemiluminescence of luminol in CFTR-inhibited vs. vehicle-treated (DMSO) neutrophils, under baseline (Fig. 4A) and inflammatory conditions (Fig. 4C), i.e. by stimulating neutrophils with a mix of the pro-inflammatory cytokine TNF-α and bacterial products fMLP, LPS or PGN. At baseline and upon priming with TNF-α, increased ROS production was observed in vehicle-treated neutrophils for all stimuli (Fig. 4A, C). On the other hand, in neutrophils treated with inh-172, ROS levels did not increase upon stimulation with fMLP, LPS and PGN, leading to an overall significant reduction in ROS levels upon CFTR inhibition compared to DMSO-treated neutrophils (Fig. 4A, C). By contrast, PwCF and healthy donors had similar ROS levels regardless of the stimuli and regardless of priming with TNF-α (Fig. 4B, D). Interestingly, two distinct populations were observed in the PwCF samples, one with class I *CFTR* mutations only showing a reduction in ROS levels, the other with class II or class I/II mutations with no reduction in ROS (*Suppl. Figure 4*), although the small sample size of each subgroup precludes statistical testing and warrants further investigation.

### Degranulation is reduced in PwCF neutrophils, but not upon CFTR inhibition by inh-172

**Fig. 5:**
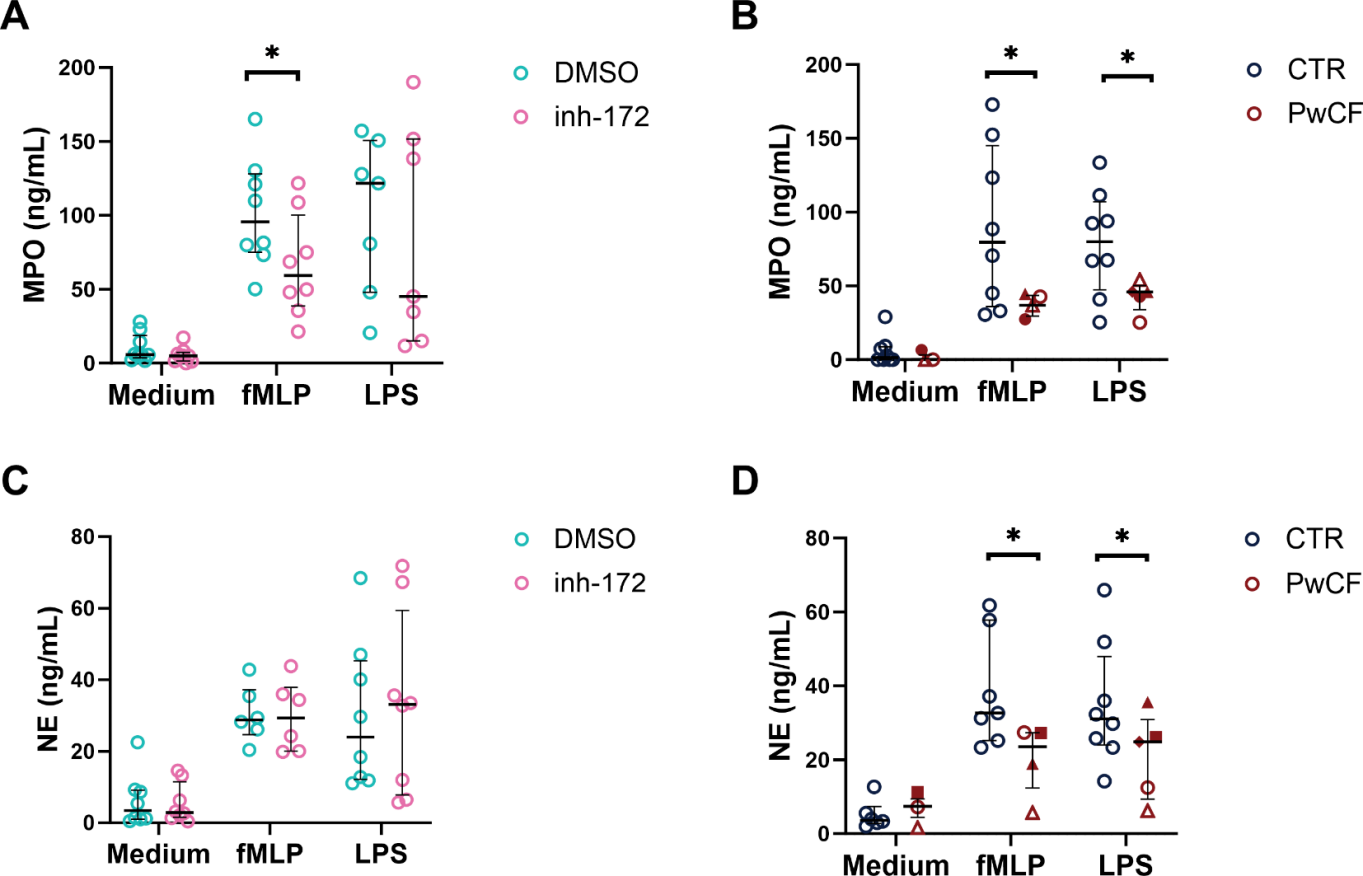
Exocytosis of myeloperoxidase and neutrophil elastase in CFTR-inhibited and PwCF neutrophils. **(A, C)** Healthy donor neutrophils were CFTR-inhibited with inh-172 or DMSO as control or **(B**,** D)** neutrophils from CF patients (PwCF) and healthy donors (CTR) were stimulated for 2 h with medium, fMLP, or LPS, whereupon the supernatant was collected and the concentration of myeloperoxidase (MPO – **A**,** B**) and neutrophil elastase (NE – **C**,** D**) was determined by ELISA. Statistical differences were determined using Wilcoxon matched-pairs signed rank test for inh-172 data and Kolmogorov-Smirnov test for PwCF data. Separation of the results according to homozygous and heterozygous groups is shown in *Suppl. Figure 5*.

To determine whether intrinsic CFTR dysfunction has implications for the release of primary granules MPO and NE by neutrophils, we induced degranulation and analyzed the conditioned media by ELISA after 2 h (Fig. 5). In the absence of stimulation (medium condition), peripheral blood neutrophils from healthy controls and CF conditions (inh-172 or PwCF) showed minimal degranulation of either MPO or NE (Fig. 5A-D). Upon stimulation with bacterial products fMLP and LPS, MPO and NE exocytosis was increased in all conditions (DMSO, inh-172 and non-CF control (CTR)) except for PwCF (Fig. 5A-D). The non-CF control group exhibited average MPO levels of around 80 ng/mL though with high donor-to-donor variability, whereas the PwCF group showed a consistent concentration of approximately 50 ng/mL with low patient-to-patient variability (Fig. 5B). This represented a significant reduction in MPO release for the PwCF vs. healthy individuals for both stimuli (fMLP and LPS). A similar reduced exocytosis response of neutrophil elastase was observed for the PwCF vs. control group (Fig. 5D). *Suppl. Figure 5* shows MPO and NE degranulation separated per mutation class. The level of reduced exocytosis was similar between class I/I, I/II and II/II. By contrast, no significant difference in the release of NE or MPO was observed for the CFTR-inhibited (inh-172) neutrophils compared to vehicle-treated neutrophils (Fig. 5A, C). In general, variability between individual donor samples was observed, in particular in the stimulated conditions (fMLP, LPS).

### Bacterial killing by neutrophils is hampered upon CFTR inhibition

**Fig. 6:**
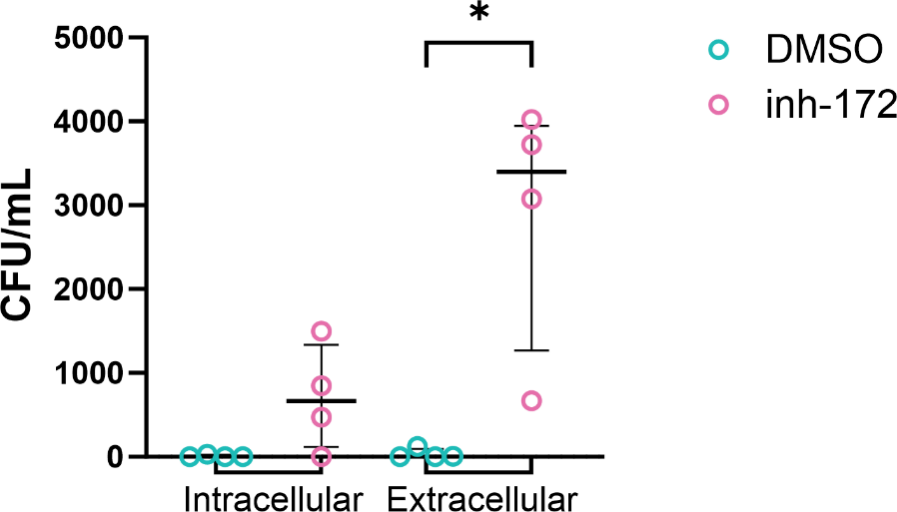
Bacterial killing by CFTR-inhibited neutrophils. Healthy donor neutrophils were CFTR-inhibited with 50 µM of inh-172 or incubated in 0.5% DMSO as control, and incubated with *E. coli* at an MOI 3 for 2 h. After incubation, the cell lysates (intracellular) and supernatants (extracellular) were plated to determine the number of bacteria surviving by quantifying the number of colony-forming units (CFU) per mL solution plated on agar plates. Statistical differences were determined using Kolmogorov-Smirnov test.

One of the major physiological functions of neutrophils is to kill bacteria. This process is the sum of the different antimicrobial functions assessed above. To assess whether the observed reduction in phagosome acidification (Fig. 2) and ROS production (Fig. 4) upon CFTR inhibition would subsequently lead to reduced bacterial killing, neutrophils were incubated with *E. coli* bacteria after pre-treatment with inh-172. Next, supernatants and neutrophil cell lysates were plated on agar plates to quantify the number of surviving extracellular and intracellular bacteria, respectively, measured as the number of colony-forming units (CFU) per mL. For the control condition (DMSO treatment), no or minimal remaining colonies were observed for either the intracellular or extracellular bacterial population, underscoring efficient bacterial killing by neutrophils with normal CFTR function. By contrast, CFTR inhibition by inh-172 notably affected the killing capacity of neutrophils, as both from the supernatants and the cell lysates more live bacteria were recovered compared to vehicle-treated neutrophils (Fig. 6). In particular, bacterial survival in the extracellular space was significantly higher for CFTR-inhibited than vehicle-treated neutrophils.

### Beyond CFTR-intrinsic defects in neutrophils: investigating the role of the CF airway micro-environment

In order to understand whether the inflamed CF lung micro-environment further affects the neutrophil effector functions shown to be deregulated upon CFTR inhibition, we set out to create an *in vitro* model to recapitulate key aspects of the human CF airways based on previously established models^14,15,23^. Specifically, we aimed to investigate the effect of the CF lung environment on the phagosome acidification during phagocytosis, which we showed was reduced upon CFTR inhibition (Fig. 2). To do so, we re-created a CF airway-like environment using a monolayer of 16HBE14o- cells, a human bronchial epithelial cell line, exposed to CF sputum supernatant or DTT (as control) along with inh-172 or DMSO for 2–3 h. In turn, neutrophils were exposed to inh-172 or DMSO for 30 min before being added on top of the epithelial monolayer. To isolate potential effects from either the airway epithelium or CF sputum supernatant on neutrophil phagocytosis, we included a ‘*no co-culture’* control without the monolayer. GM-CSF was added throughout the experiments to extend the neutrophils’ lifespan.

**Fig. 7:**
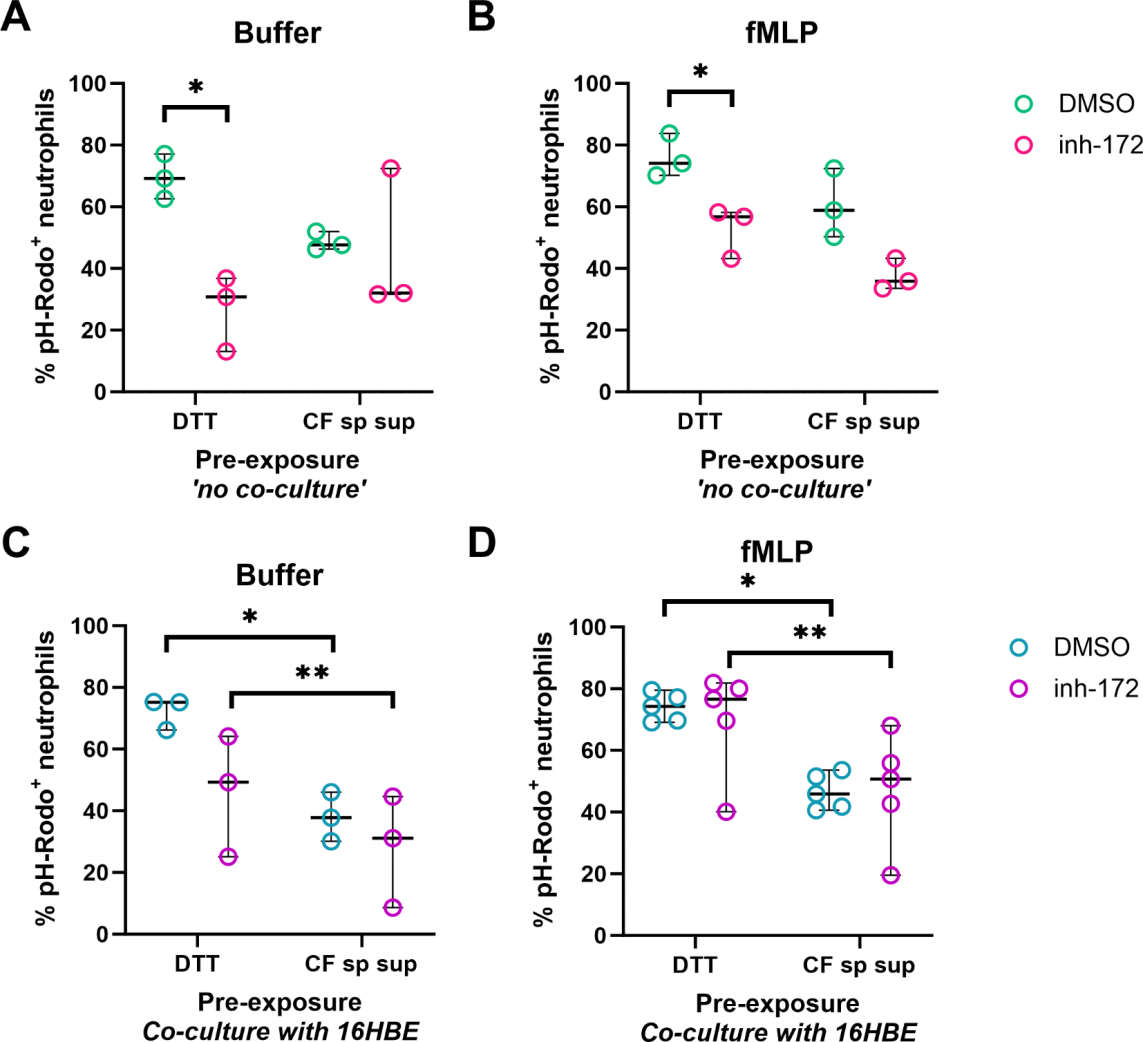
Effect of pre-exposure to CF lung micro-environmental stimuli on neutrophil phagocytosis and viability. Phagocytosis Assays **(A**,** B)**: Wells were pre-treated with either 0.02% (v/v) DTT or 20% CF sputum supernatant (CF sp sup) and then exposed to either inh-172 or DMSO for 2–3 h. Healthy donor neutrophils were pre-incubated with inh-172 or DMSO for 30 min, then added to the treated wells for 2 h. Neutrophils were collected, centrifuged, and replated for the phagocytosis assay, either primed with buffer **(A)** or fMLP **(B)** and subsequently exposed to pHrodo^TM^-labelled *S. aureus* bioparticles for 3 h. Co-culture and Phagocytosis Assays **(C**,** D)**: Epithelial cells were incubated with 0.02% (v/v) DTT or 20% CF sputum supernatant (CF sp sup) and either inh-172 or DMSO for 2–3 h. Healthy donor neutrophils, pre-incubated with inh-172 or DMSO for 30 min, were then added to the epithelial cell cultures for 2 h. After co-culture, neutrophils were collected, centrifuged, and replated for the phagocytosis assay. Neutrophils were primed with either buffer **(C)** or fMLP **(D)** and exposed to *S. aureus* bioparticles for 3 h. Phagocytosis was assessed by imaging and calculating the percentage of pHrodo-positive cells. Statistical differences were determined using paired t tests. Abbreviations: DTT: Dithiothreitol; CF sp sup: cystic fibrosis sputum supernatant.

First, to exclude potential effects of DTT (in view of the fact that the sputum supernatant was collected upon DTT treatment), we compared the degree of phagocytosis under DTT and buffer (PBS) pre-treatment. No differences in phagocytosis levels were observed between the DTT and buffer conditions in the *no co-culture* control (*Suppl. Figure 6 A*,* B*), and neither in the 16HBE14o- co-culture of neutrophils treated with inh-172 (*Suppl. Figure 6 C*). Only in vehicle-treated neutrophils an increase in phagocytosis was observed after DTT pre-treatment (*Suppl. Figure 6 C*,* D*). In line with our previous observations (Fig. 2), inh-172 treatment led to an approximately 2-fold reduction of *S. aureus* pH-Rodo positive neutrophils compared to DMSO control, indicating impaired phagosome acidification in the DTT control ‘*no co-culture’* condition with and without fMLP induction of phagocytosis (Fig. 7A, B). Upon pre-incubation with CF sputum supernatant, the difference between DMSO and inh-172 was diminished to ~ 1.5-fold, but mainly because of a reduction in phagocytosis in the vehicle-treated neutrophils (~ 1.4-fold decrease for CF sputum supernatant vs. DTT condition, Fig. 7A). This reduction was even more pronounced when neutrophils were co-cultured with 16HBE14o- cells (~ 2-fold decrease for CF sputum vs. DTT, Fig. 7C, D). Surprisingly, co-culture with epithelial cells abrogated the inh-172 induced reduction in phagocytosis, in particular in the fMLP stimulated condition (Fig. 7D). To exclude a possible adverse effect of 16HBE14o- co-culture or CF sputum supernatant treatment of neutrophils, viability was evaluated during the co-culture experiments using Sytox Green to visualize and quantify the amount of cell-free DNA. Neutrophil viability was maintained at comparable levels between DTT and sputum treatment (*Suppl. Figure 6E*), indicating that the epithelial monolayer did not adversely affect neutrophil survival. We included PMA as a positive control, which increases cell-free DNA due to NETosis of treated neutrophils. Indeed, we observed more cell-free DNA upon PMA treatment, in particular in neutrophils treated with inh-172 (*Suppl. Figure 6 F*).

## Discussion

Cystic fibrosis (CF) is caused by CFTR dysfunction which leads to imbalanced ion homeostasis and recurrent bacterial infections in the airways, accompanied by chronic neutrophilic inflammation^24^. While CFTR modulators have significantly increased lung function and reduced exacerbations and hospitalizations^25^, their ability to resolve neutrophil-driven inflammation is still uncertain. In particular, it is known that the chronic presence of neutrophils in the airways poses a significant risk of irreversible damage by the secretion of many potent enzymes with matrix-degrading capacity. Hence, it remains critical to understand which therapeutic options are available to limit the damaging effects of chronic neutrophil presence in the CF airways.

The expression of CFTR in neutrophils remains a subject of debate. CFTR levels have been found to vary from undetectable^13,26^ to localized in the membrane of phagosomes^27^. CFTR protein has been shown to be upregulated after PMA stimulation and recruitment^28^. Painter *et al.* have established that neutrophils use pre-synthesized and pre-stored CFTR during microbial killing^12^. Our samples were generated without any stimulation of neutrophils and, therefore, reflect the levels of CFTR detected at baseline. In our study, nested PCR showed low levels of *CFTR* mRNA levels in primary human neutrophils, likely due to high levels of RNases and the low per-cell RNA content^29^. In healthy donors, we observed varying levels of CFTR staining intensity by immunocytochemistry, but overall, staining appeared specific when compared to a CF control homozygous for a class I mutation (G542X), where no signal was detected. These findings support a certain degree of CFTR expression in primary neutrophils. Since the healthy donors in our study were not genetically screened for potential mutations in *CFTR*, we cannot exclude interference of carrier status with CFTR staining intensity in this cohort^30^.

Next, we investigated which neutrophil effector functions were affected by a dysfunctional CFTR protein. We attempted to create a new model to answer these questions by treating healthy donor neutrophils with inh-172, a chemical that selectively inhibits CFTR channel function^20^. Inh-172 has been reported both for acute and chronic inhibition of CFTR activity^21,31,32^. We hypothesized that proteins interacting with CFTR would not be affected due to the short duration of the inh-172 treatment in the different assays (maximum 3–6 h), allowing us to dissect directly the role of CFTR. PwCF neutrophils were included as a control where presumably functional impairments would relate both to CFTR and its interactome.

The most likely functional process affected directly by the absence of CFTR is phagocytosis since CFTR is one of the main channels providing chloride ions for the acidification of the phagosome^12,21^. Indeed, we observed that treatment with inh-172 resulted in reduced acidification. This is in line with the observed increased alkalinity in CF neutrophil phagosomes reported by others^33^. Previous research using inh-172 on neutrophils translated to a reduction of around 45% in iodide uptake by the phagosomes as a measure for CFTR ion transport activity^21^. Iodide is a halide, not naturally present in a cell, that is used to assess specifically CFTR permeability since other anion co-transporters will not be permeable^34^. Next, we assessed the acidification capacity of phagosomes in peripheral blood neutrophils from a small cohort of PwCF (*n* = 6) to corroborate the obtained inh-172 data. However, no significant change in phagocytosis capacity was observed for PwCF neutrophils, which has also been reported by others^35^. Residual CFTR activity might explain this; however, in our study, we only included PwCF carrying either class I or II mutations, which typically have minimal to no baseline function. When separating the PwCF samples per mutation class, we observed that mainly neutrophils from PwCF homozygous for class II mutations showed reduced phagocytosis, relative to the control, and with comparable ratios as observed in CFTR-inhibited neutrophils^36^. However, the small sample size for these subdivisions warrants future investigation for statistically supported conclusions. Additionally, we observed high variability in response for both the control and PwCF group, which could be due to differences in gender and age (which we matched as closely as possible), circadian cycle regulation, among others, that could have been amplified by the current small sample size.

Other functional defects have been reported in CF neutrophils. For example, Robledo-Avila *et al.* reported reduced ROS, NETosis and bacterial killing^37^. Their assessment of ROS focused on a narrower range of ROS molecules compared to our study, in which we additionally measured H_2_O_2_ and superoxide levels^38^. Neutrophils treated with inh-172 had similar values to vehicle-treated at baseline, while, upon stimulation with fMLP, LPS or PGN, ROS production decreased. This suggests that CFTR inhibition may impair ROS production under inflammatory and bacterial conditions. A more recent study looking at an identical CF population as Robledo-Avila *et al*., observed no difference in ROS production upon stimulation with fMLP and LPS and an increase only with PMA and opsonized zymosan^23^. Our study in a CF adult population aligns with these results as likewise, no significant difference in ROS production was observed. When subdividing the PwCF data into class I/ I, class I/II and class II/II however, neutrophils carrying two class I mutations tended to produce less ROS upon TNF-α priming. This aligns with the inh-172 data, although a bigger sample size is required to substantiate these observations, in particular in light of the high variability in the control group.

PwCF neutrophils showed impaired NE and MPO exocytosis upon stimulation with fMLP and LPS. At first sight, this seems to contradict the observations that CF airways contain elevated levels of proteases and reduced levels of protease inhibitors^8,39^. However, although reduced, degranulation was not fully impaired. Hence, the high numbers of infiltrating neutrophils in the CF airways may compensate for the observed reduced degranulation per individual neutrophil. Furthermore, neutrophils typically get entrapped in the thick CF mucus with delayed apoptosis, which can lead to increased release of neutrophil content^40,41^. While the actual exocytosis ultimately determines the efficiency of bacterial killing, it would be interesting to explore whether the amount of primary granule material, containing MPO and NE, is reduced in PwCF neutrophils. The observation that degranulation was not reduced in CFTR-inhibited neutrophils, suggests a direct or indirect involvement of CFTR during granule synthesis, which already occurs in the bone marrow-residing progenitor cells. Pohl *et al*. reported that impaired degranulation of secondary and tertiary granules likely finds its origin not in impaired granule formation, but in its defective trafficking^42^. Indeed, a normal NE amount has been reported in CF neutrophil lysates^43^, warranting further studies on progenitor cells to better understand the involvement of CFTR in granule formation, trafficking and exocytosis. Besides NE and MPO release through degranulation, these enzymes can also be released through NETosis. High extracellular levels of MPO have been shown to trigger additional degranulation in neutrophils^44^. This suggests that if high levels of MPO are released due to NETosis, CF neutrophils may release more MPO in a feed-forward loop and overcome the initial CFTR dysfunction.

To functionally validate the observed intrinsic defects in CFTR-inhibited neutrophils in phagocytosis and ROS production, we performed a bacterial killing assay. While *Escherichia coli* is not a primary pathogen in CF^45^, it serves as an effective model to study the impact of CFTR inhibition on neutrophil bacterial killing, in particular since several studies have detected *E. coli* in PwCF lungs^45,46^. Previous studies, such as the one from Painter *et al.* reported reduced bacterial killing of *Pseudomonas aeruginosa* of neutrophils treated with the CFTR inhibitor, GlyH-101^21^, or upon inhibition of NADPH oxidase and MPO. They concluded that the killing could be CFTR- and oxidant-dependent, but also independent. Another study showed reduced killing of bacterial isolates from CF airways, although no impairment was found in phagocytosis^47^. Our results for *E. Coli* align with these findings and support that CFTR inhibition using inh-172 also compromises the bactericidal activity of neutrophils as evidenced by increased survival of bacteria. Coupling these observations back to the intrinsic defects we measured in CFTR-inhibited neutrophils, the reduced phagosome acidification aligns most closely to reduced intracellular killing. It is not possible, however, to link the reduced ROS production detected by luminol with intra- or extracellular killing, as this probe is not specific for one or the other compartment^48,49^. Increasing the sample size, besides repeating the bacterial killing with more CF-relevant bacterial strains would be needed for further confirmation of our findings. Overall, our results suggest that CFTR dysfunction affects neutrophil effector functions and leads to reduced bacterial killing. Therapies aiming to restore mutant CFTR protein processing and function are thus expected to improve the neutrophil defects observed. Indeed, CFTR modulator treatment in PwCF has been shown to improve CF neutrophil responses when analyzed *ex vivo*^33,42,50^, although these studies did not look at the treatment response compared to the individual’s baseline. Future studies on CFTR targeted therapies could thus be conducted in the inh-172 CFTR dysfunction model to provide a better understanding of their positive effect on specific neutrophil functions compared to a matched baseline control from the same donor.

So far, we dissected potential intrinsic defects of CFTR dysfunctional neutrophils using *in vitro* assays in the absence of confounding factors such as the CF airway epithelium or inflammation. However, the inflamed CF airway environment could heavily impact the net CF neutrophil effector function. Indeed, CF airway and blood neutrophils present distinct transcriptional profiles, as do healthy blood neutrophils exposed to a CF airway environment, altering their metabolic and adaptative mechanisms and decreasing antimicrobial processes^14^. We therefore opted to co-culture neutrophils with CF sputum supernatant and/or airway epithelial cells in order to recreate an inflammatory CF airway environment *in vitro*. Our results showed that healthy (vehicle-treated) neutrophils displayed reduced phagocytosis levels upon exposure to CF sputum, in particular when co-cultured with 16HBE14o- airway epithelial cells. CF sputum-induced impairments in healthy neutrophils have previously been described^14,15,51^. We hypothesize that reduced phagocytosis might be the result of upregulation of other cellular processes upon stimulation with the multitude of pro-inflammatory and bacterial stimuli present in CF sputum, or even exhaustion. Interestingly, the impaired phagosome acidification we observed upon CFTR inhibition through inh-172, was reduced or even abolished when neutrophils were co-cultured with CF sputum without or with 16HBE14o- cells, respectively. While we cannot exclude that inh-172 might have been bound to the CFTR channels present in the 16HBE14o- cells, a high (50 µM) concentration of inh-172 was maintained throughout the experiments. Alternatively, other chloride channels contributing to phagosome acidification might have been activated, to take over the role of inhibited CFTR. We confirmed that co-culturing neutrophils with epithelial cells and CF sputum supernatant for 2 h did not reduce the neutrophil viability, in line with previous work^47^. One of the limitations of our study was the short exposure time to CF sputum and epithelial co-culture. Future experiments should focus on extending the co-cultures, besides adding neutrophils at consecutives moments, as this will more closely mimic chronic neutrophil infiltration in the CF airways and hence allow a better understanding of the consequences of this exposure to the epithelium.

In conclusion, we set up a CF-like in vitro model using CFTR inhibitor inh-172, to induce CFTR dysfunction in healthy neutrophils. This allowed to link impairments in phagocytosis and ROS production to CFTR inhibition, which functionally led to reduced bacterial killing. The inh-172-induced CFTR dysfunction model provides an interesting alternative to probe the role of CFTR in immune cell function, as donor-to-donor variability between groups is abolished. Indeed, we generally observed high donor-to-donor variability both in the control and PwCF groups, affecting for some assays a robust statistical confirmation of obtained differences with the given sample sizes used. While our study aimed at modelling the behavior of PwCF neutrophils through acquired CFTR dysfunction using inh-172, future studies could explore the effect of cigarette smoke on neutrophil functions in the context of other chronic lung diseases such as chronic obstructive pulmonary disease, given its ability to inhibit CFTR function in the epithelium of people without *CFTR* mutations^52^.

Our study thus suggests CFTR contributes to neutrophil-mediated pro-inflammatory responses and bacterial clearance, despite its low expression, and that loss of CFTR function affects these functions. The altered response of CFTR-inhibited neutrophils co-cultured with CF sputum supernatant and airway epithelial cells emphasizes the complexity of neutrophil responses in the context of their presence in an inflamed and infected CF airway environment. Our findings therefore could contribute to an increased understanding of which neutrophil responses might benefit from CFTR-targeted therapies, and which functions likely require other therapeutic avenues. Irrespectively of the strategy, it is imperative to reduce the chronic neutrophil-driven inflammation in PwCF and even broader, other chronic lung diseases suffering from similar neutrophilic processes, as their chronic presence most likely causes more harm than benefit, leading to progressive tissue destruction if left untreated.

## Electronic supplementary material

Below is the link to the electronic supplementary material.

Supplementary Material 1

## Data Availability

Raw data are available upon request to the corresponding author.
