# Supplementary material for: Inhibiting CFTR through inh-172 in primary neutrophils reveals CFTR-specific functional defects
Source: Sci Rep. 2024 Dec 28;14:31237. doi: 10.1038/s41598-024-82535-z (PMC11682091; doi:10.1038/s41598-024-82535-z)
Supplement: Supplementary file 1 — Supplementary Material 1 [file 41598_2024_82535_MOESM1_ESM.docx]

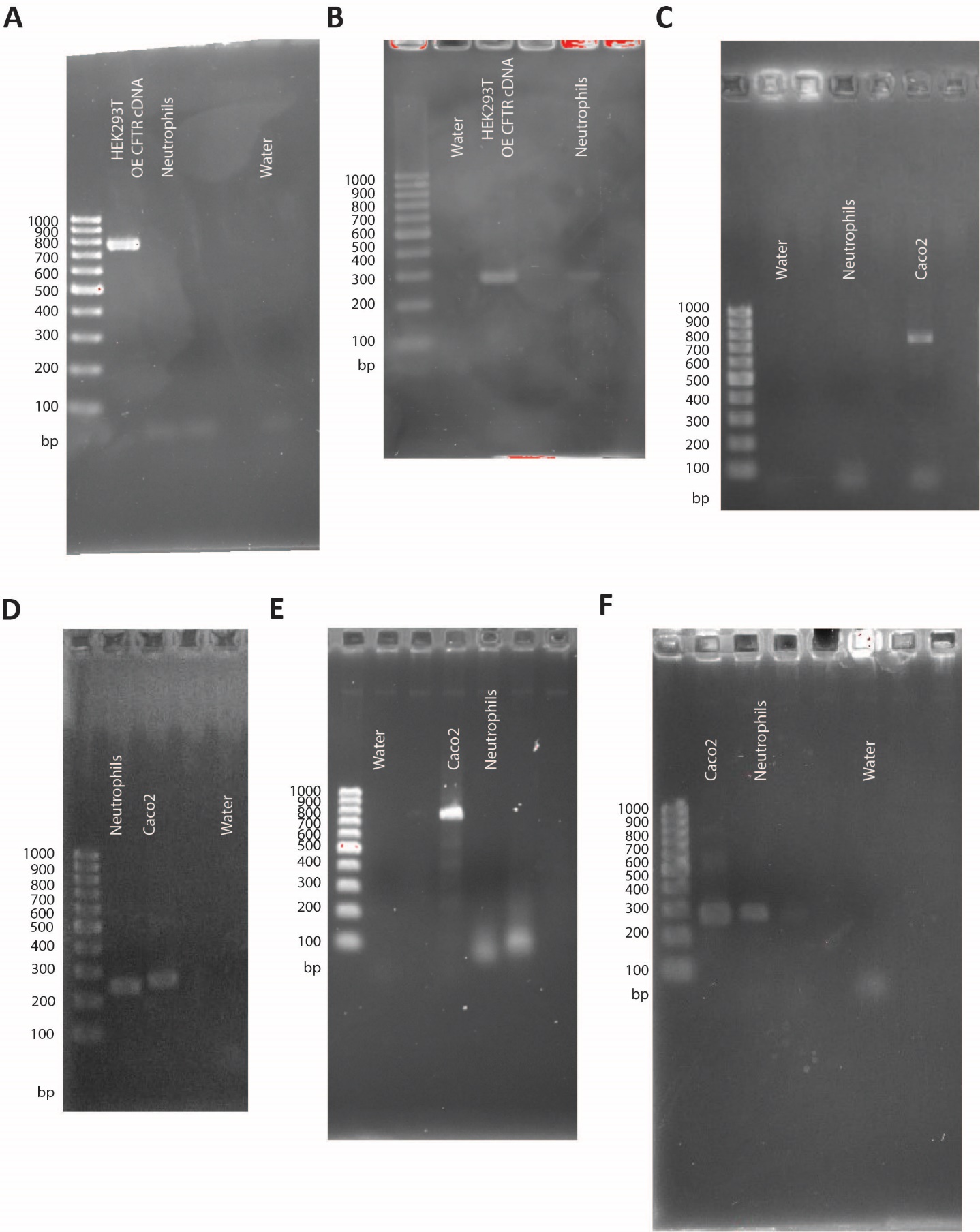


**Supplementary figure 1. Detection of CFTR expression in primary non-CF neutrophils using nested PCR**. (A, B) Original gel images from Fig. 1B-C, respectively, of non-CF individual (annotated sample 1). (C, E) *CFTR* cDNA from neutrophils of two non-CF individuals (labelled non-CF 2 and 3, respectively), Caco2 (*positive control*) and water (*negative control*) were amplified using Taq Polymerase in the first round of PCR (PCR1). (D, F) A second PCR (PCR2) was performed on the purified PCR1 amplicon.

**
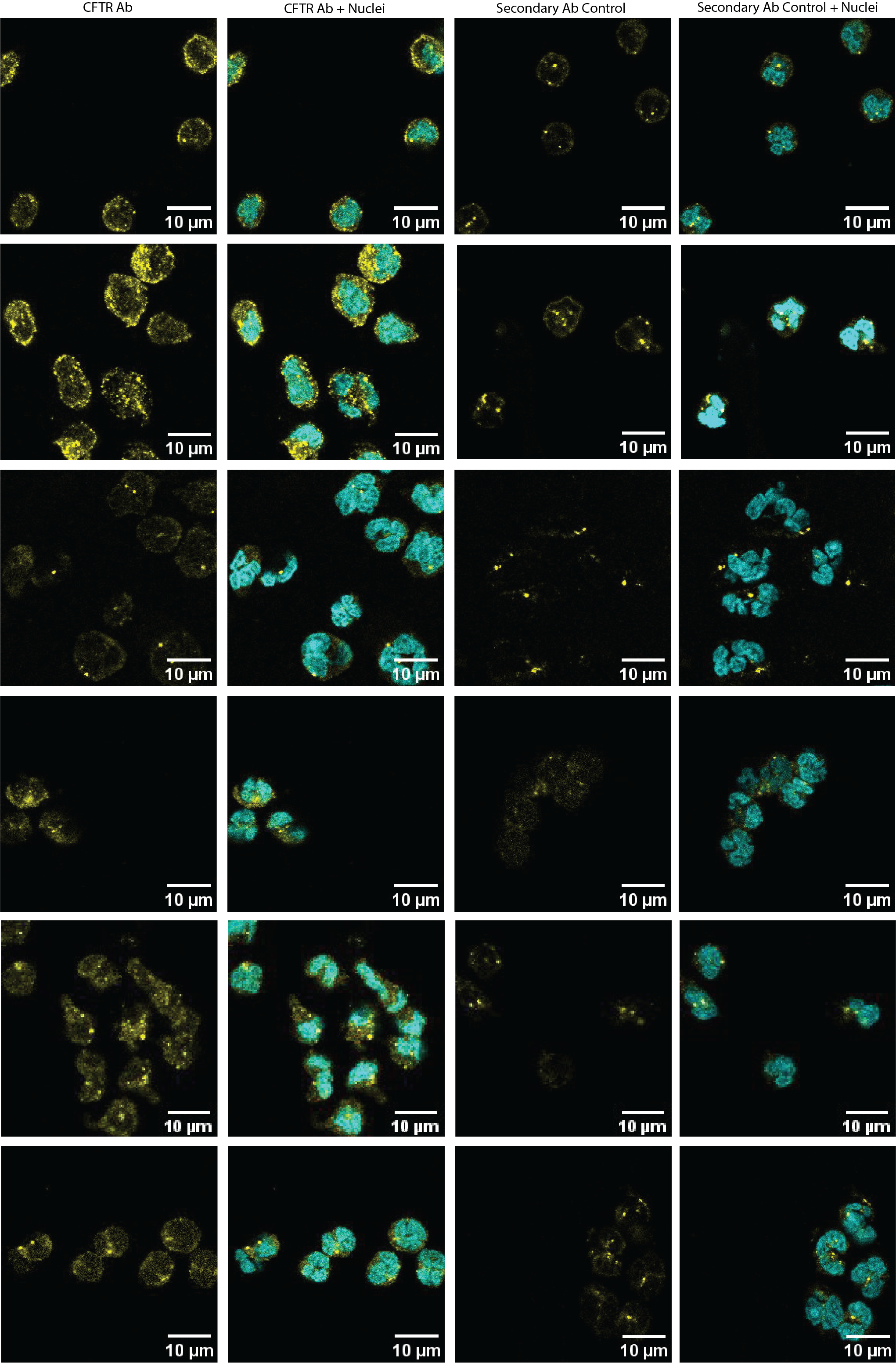
Supplementary figure 2. Detection of CFTR expression by immunocytochemistry.** Immunofluorescence images of primary neutrophils from 6 healthy donors (one per row). Samples were stained for CFTR (yellow) and counterstained with the nuclear stain DAPI (cyan) in the first two columns. The last two columns show immunofluorescence from the secondary antibody only, serving as a control in the third column, and combined with DAPI in the fourth column. Images from six additional healthy donors are provided in Supplementary Figure 2. The scale bar represents 10 µm.


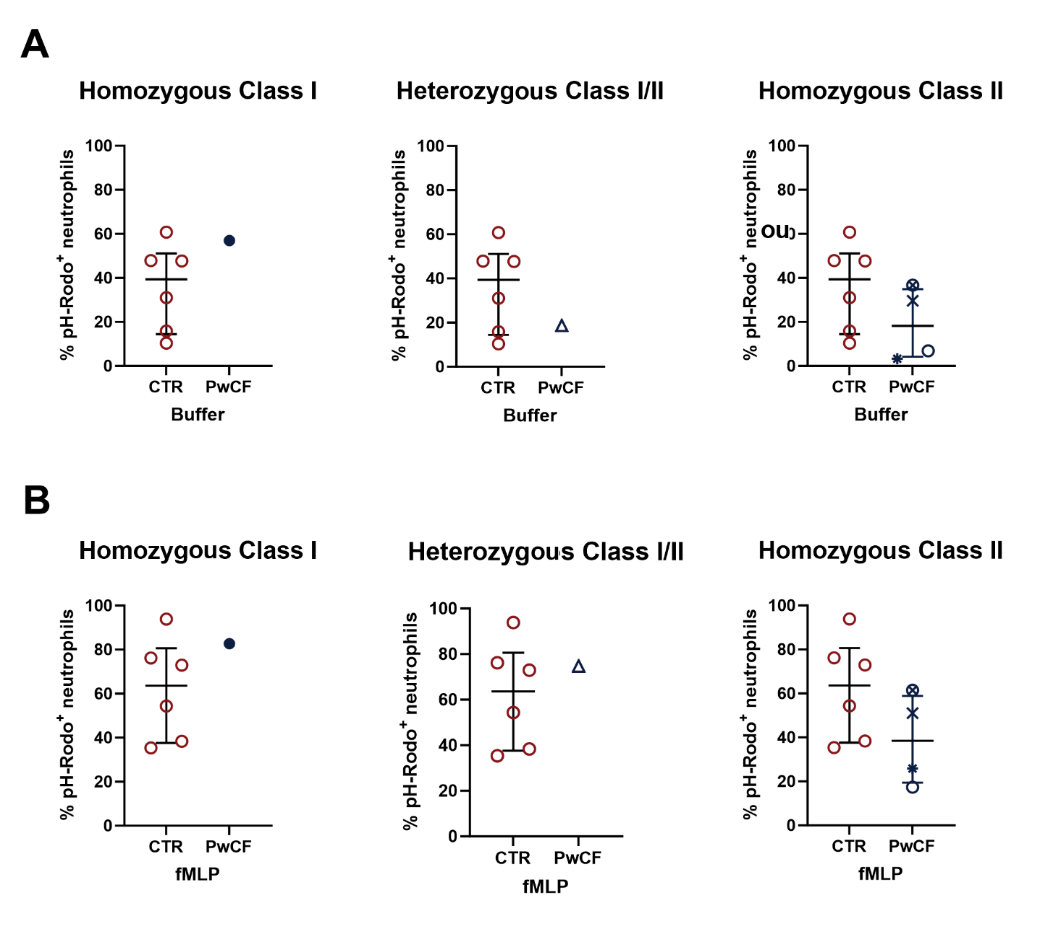


**Supplementary figure 3. Phagocytosis of PwCF neutrophils separated by mutation classes.**Neutrophils from healthy donors or PwCF were labelled with calcein, whereupon the cells were primed for 10 min with buffer (A) or fMLP (B) and exposed to either *Staphylococcus aureus-*conjugated pHrodo-labeled bioparticles. The cells were subsequently microscopically imaged for 2-3h. Data for class I homozygous are presented in the first panels, heterozygous with class I and class II mutations are presented in the middle panels and class II homozygous are presented in the last panels. Each data point is the average of percentage of pHrodo positive cells of all images after 3h of incubation with *S. aureus*-conjugated bioparticles. Graphs represent the data as median ± interquartile range. Statistical differences were determined using Kolmogorov-Smirnov test.


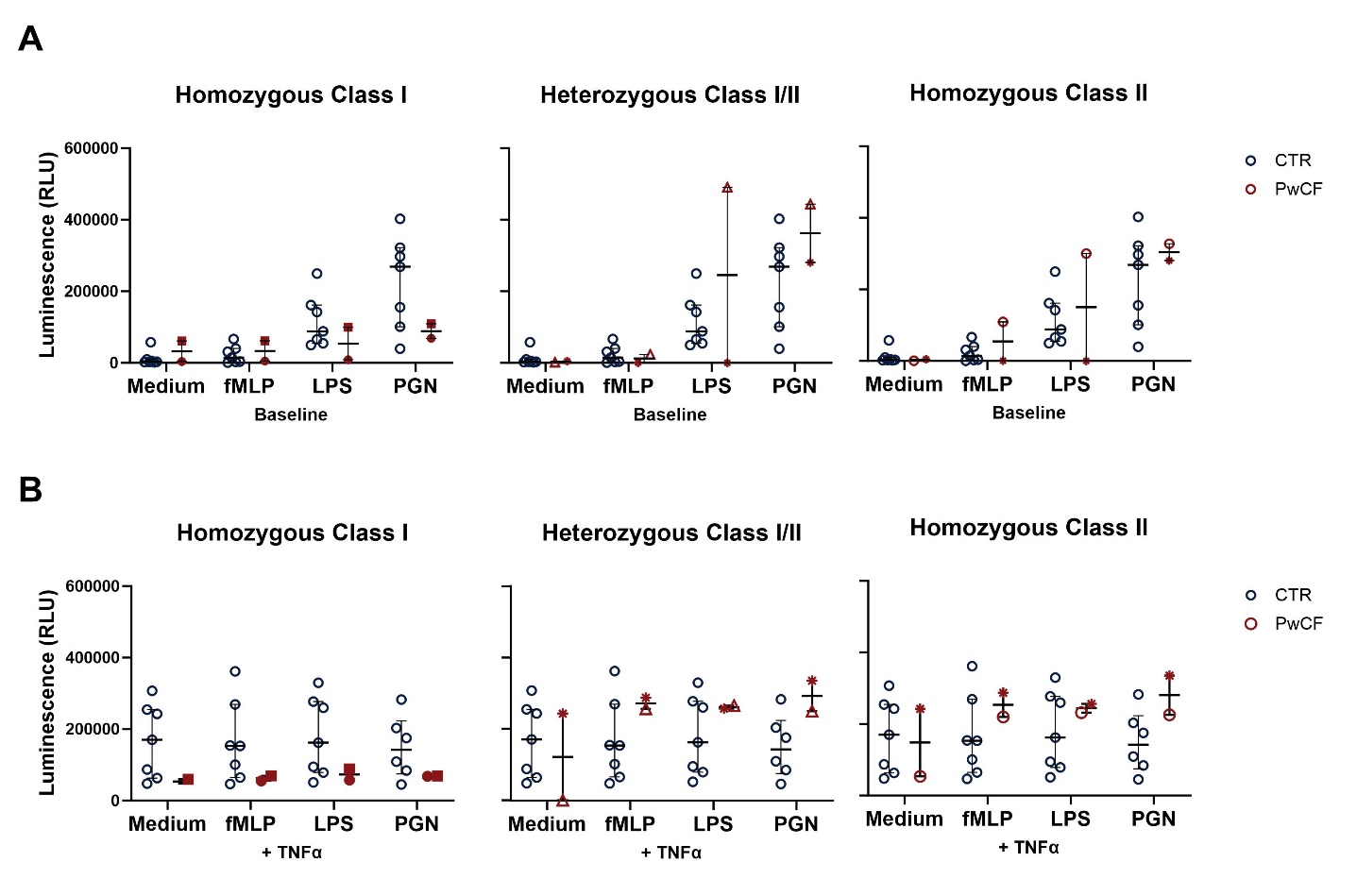
**Supplementary figure 4. ROS production of PwCF neutrophils separated by mutation classes.** Total reactive oxygen species (ROS) production, quantified using a chemiluminescence-based assay, of neutrophils from healthy donors (CTR) or people with cystic fibrosis (PwCF) was induced with medium, N-formyl-methionyl-phenylalanine (fMLP), Lipopolysaccharides (LPS) or peptidoglycan (PGN). Tumor Necrosis Factor-alpha (TNFα) was added as a priming agent to enhance neutrophil function. Peak luminescence is shown for Medium, fMLP, LPS or PGN. (A) Baseline ROS production without TNF-α for PwCF compared to CTR. (B) ROS production with TNF-α priming for PwCF compared to CTR. Graphs represent the data as median ± interquartile range. Statistical differences were determined using Kolmogorov-Smirnov test. RLU: relative light units.


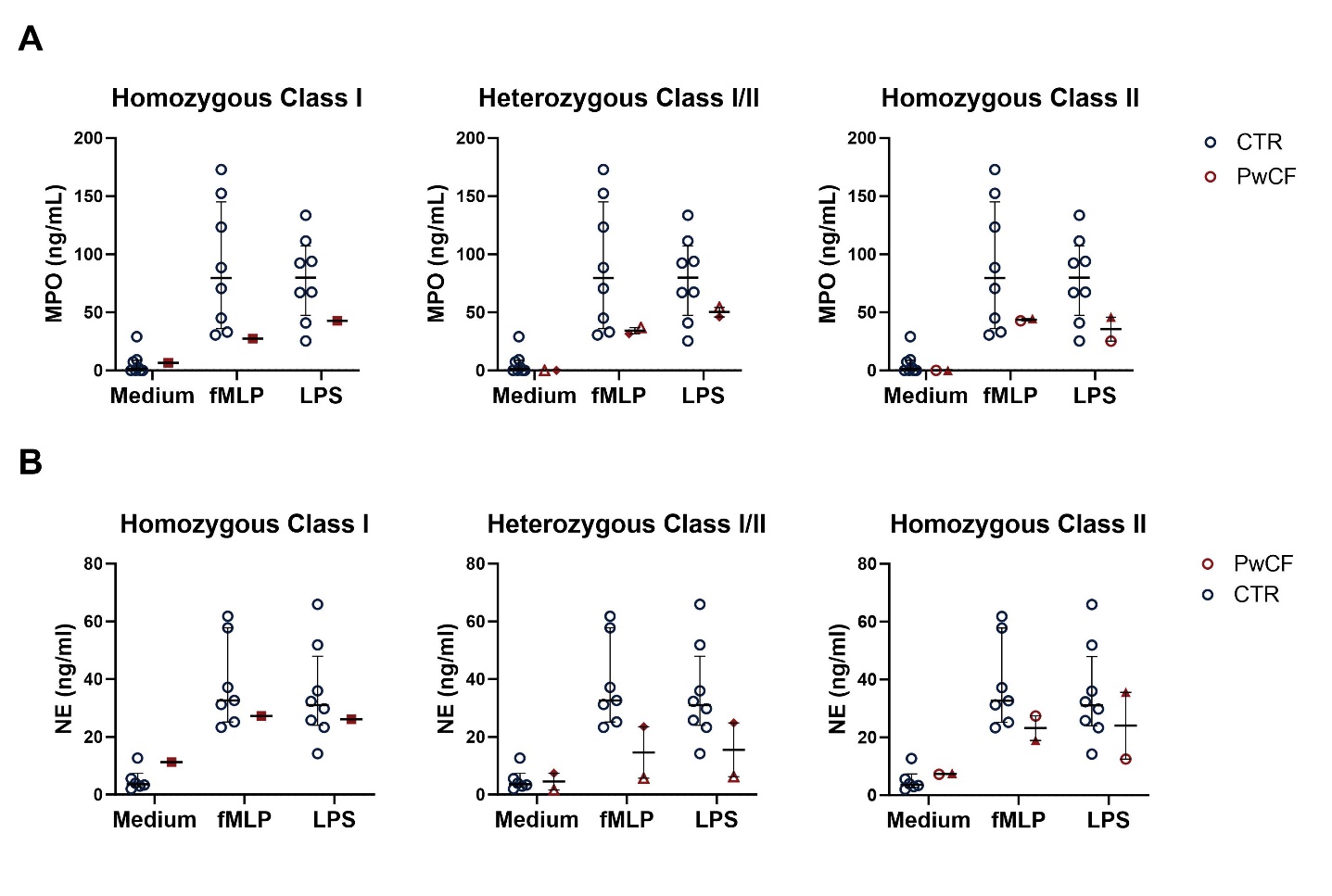


**Supplementary figure 5. Exocytosis of NE and MPO of severe PwCF neutrophils separated by mutation classes.** Neutrophils from CF patients (PwCF) or healthy donors (CTR) were stimulated for 2 h with medium, fMLP and LPS, whereupon the supernatant was collected and the concentration of myeloperoxidase (MPO) was determined by ELISA. (A) MPO concentration. (B) NE concentration.


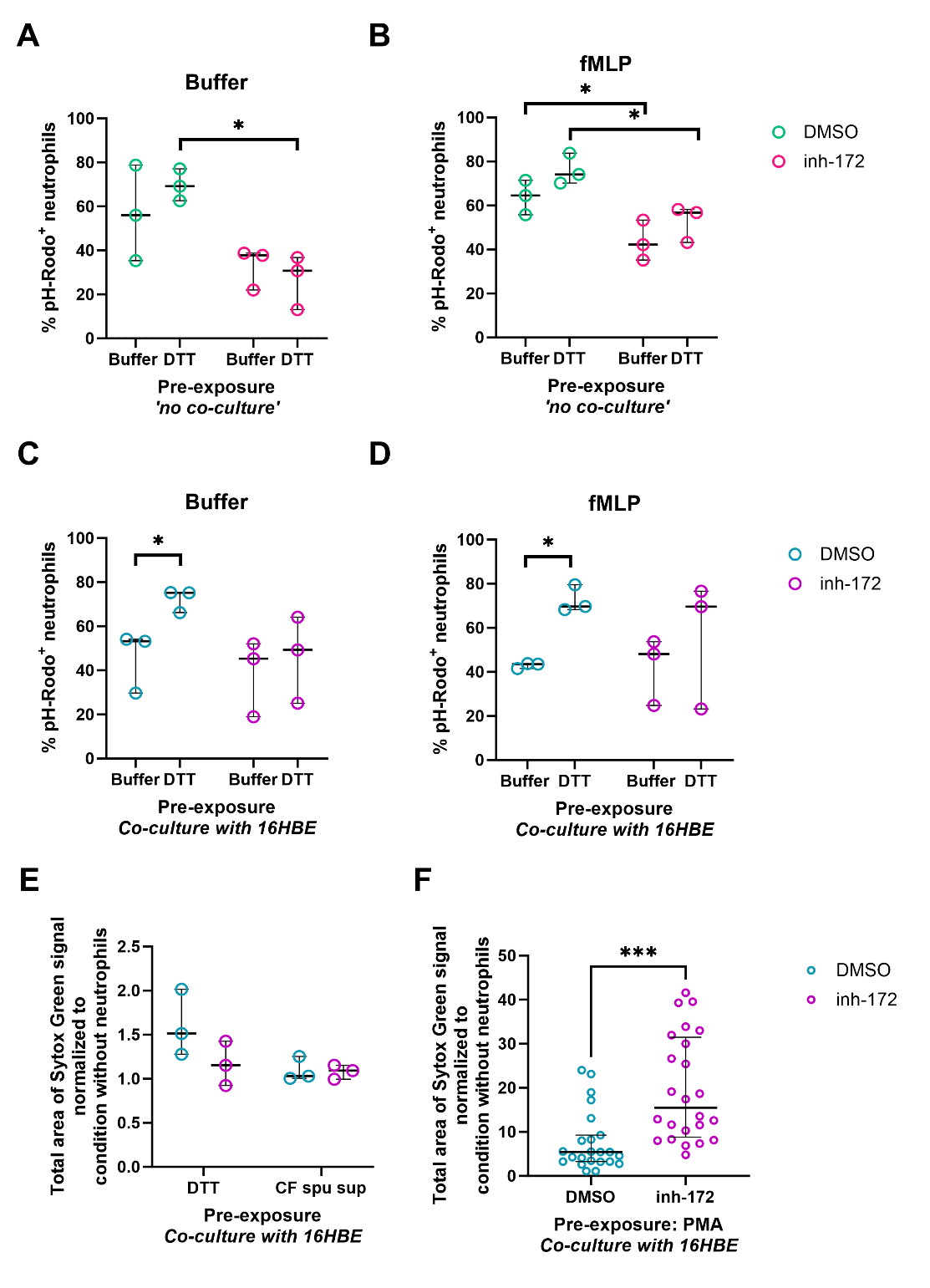


**Supplementary figure 6. Effect of pre-exposure to the cystic fibrosis (CF) environment on neutrophil phagocytosis and viability.** Phagocytosis Assays (A, B): Wells were pre-treated with either 0.02% (v/v) DTT or 0.02% (v/v) Buffer (PBS) and then exposed to either inh-172 or DMSO (vehicle control) for 2-3 hours. Healthy donor neutrophils were pre-incubated with inh-172 or DMSO for 30 minutes, then added to the treated wells for 2 h. Neutrophils were collected, centrifuged, and replated for the phagocytosis assay, either primed with buffer (A) or fMLP (B) and subsequently exposed to S. aureus-conjugated pHrodo-labeled bioparticles for 3h. Co-culture and Phagocytosis Assays (D, E): Epithelial cells were incubated with 0.02% (v/v) DTT or or 0.02% (v/v) Buffer (PBS) and either inh-172 or DMSO for 2-3 hours. Healthy donor neutrophils, pre-incubated with inh-172 or DMSO for 30 minutes, were then added to the epithelial cell cultures for 2 hours. After co-culture, neutrophils were collected, centrifuged, and replated for the phagocytosis assay. Neutrophils were primed with either buffer (C) or fMLP (D) and exposed to S. aureus-conjugated pHrodo-labeled bioparticles for 3 h. Phagocytosis was assessed by imaging and calculating the percentage of pHrodo-positive cells. Statistical differences were determined using the Wilcoxon matched-pairs signed rank test. Statistical differences were determined using paired t tests. Viability Assays (D): Free DNA in the co-culture was labelled with Sytox Green to assess neutrophil viability for exposure to DTT, CF sputum sup (CF spu sup) (E) and PMA (F). Images were taken after 2 hours of culture. The total area of Sytox Green fluorescence was normalized to control conditions without neutrophils. (E) Graph represents the median values from 3 donors. Statistical differences were determined using paired t tests. (F) Graph represents the values for individual images from 3 donors. Statistical differences were determined using Kolmorov- Smirnov test.

**Supplementary File 1: Segmentation of Cells.** This file contains a script designed for batch processing of fluorescent images to segment cells, analyse their areas, and save the results. Written in ImageJ Macro language (.ijm file), this script automatically creates binary images using an automatic threshold, watershed segmentation, and particle analysis. The resulting segmented images and data are exported to an output directory.

//To be read in ImageJ with Language ImageJ Macro

// Set the input and output directories

dir = getDirectory("Choose a Directory");

//Saves individual measurements for each image

outputDir = getDirectory("Choose an Output Directory for Original Segmentations");

//Saves resultant images

maskOutputDir = getDirectory("Choose an Output Directory for Masks");

// Get the list of files in the input directory

list = getFileList(dir);

// Set measurements to only area

run("Set Measurements...", "area redirect=None decimal=3");

// Loop over all files in the folder

for (i = 0; i < list.length; i++) {

if (endsWith(list[i], ".tif") || endsWith(list[i], ".jpg") || endsWith(list[i], ".png")) {

open(dir + list[i]);

run("8-bit");

run("Duplicate...", " ");

// Enhance brightness and contrast

run("Enhance Contrast", "saturated=0.35");

// Open the threshold adjustment dialog

run("Threshold...");

//waitForUser("Adjust the threshold and apply it. Then press Enter to continue.");

// Convert to mask after user has adjusted the threshold

run("Convert to Mask");

// fill holes

run("Fill Holes");

// Apply watershed

run("Watershed");

// Analyze particles - replace 'minSize' and 'maxSize' with your size settings

minSize = 100;

maxSize = 800;

run("Analyze Particles...", "size=" + minSize + "-" + maxSize + " exclude clear include summarize add show=Masks");

// Save the mask image

saveAs("Tiff", maskOutputDir + replace(list[i], ".tif", "_mask.tif"));

// Create a mask from the particle analysis

//run("Create Mask");

maskTitle = getTitle(); // Get the title of the mask window

// Save the results as CSV

saveAs("Results", outputDir + replace(list[i], ".tif", ".csv"));

// Clear ROI manager and results

roiManager("Deselect");

roiManager("Delete");

// Close all windows except the summary

close("*");

}

}

// Finished

print("Batch processing completed!");

**Supplementary File 2: Compositive image generation from red and green channels.** This script performs batch processing of images contained in two directories, each containing either the red and green channels. Image contrast is enhanced and the channels are merged into an RGB image, which is saved in an output directory. Developed in ImageJ Macro language (.ijm file).

//To be read in ImageJ with Language ImageJ Macro

// Set the input directories for the red and green channel images

redDir = getDirectory("Choose Directory of Red Channel Images");

greenDir = getDirectory("Choose Directory of Green Channel Images");

outputDir = getDirectory("Choose Output Directory for Composite Images");

// Get the list of red files

redFileList = getFileList(redDir);

// Initialize a log in the console

print("Composite Image Creation Log\n\n");

//Assumes files have the same name

for (i = 0; i < redFileList.length; i++) {

if (endsWith(redFileList[i], ".tif") || endsWith(redFileList[i], ".tiff")) {

// Remove the "redcal" prefix from the red channel image name

originalRedFileName = redFileList[i];

// Extract the base name of the image

baseName = substring(redFileList[i], 0, lastIndexOf(redFileList[i], "."));

print("Processing: " + baseName);

// Construct the corresponding green channel image path

greenFile = greenDir + redFileList[i];

// Check if the green channel image exists by attempting to open it

greenImageExists = false;

list = getFileList(greenDir);

for (j = 0; j < list.length; j++) {

if (list[j] == redFileList[i]) {

greenImageExists = true;

break;

}

}

// If a matching green channel image is found, proceed with the merging

if (greenImageExists) {

print("Found matching green image: " + greenFile);

// Open the red channel image using the original file name with "redcal" prefix

open(redDir + originalRedFileName);

titleRed = getTitle();

run("Enhance Contrast", "saturated=0.35");

run("Duplicate...", "title=red");

// Open the green channel image

open(greenFile);

titleGreen = getTitle();

run("Enhance Contrast", "saturated=0.35");

run("Duplicate...", "title=green");

// Merge the channels

run("Merge Channels...", "c1=red c2=green create keep");

// Convert the composite to RGB

run("Make Composite");

run("RGB Color");

// Save the composite image

saveAs("Tiff", outputDir + baseName + "_composite.tif");

print("Saved composite image: " + baseName + "_composite.tif");

// Close all images

close("red");

close("green");

close(baseName + "_composite");

close("*");

} else {

print("No matching green image found for: " + redFileList[i]);

}

}

}

**Supplementary File 3: Segmentation of possible phagocytosis regions.** The script processes RGB images to identify regions of overlapping green and red signals, which are indicative of phagocytosis (*yellow* regions). Images are split into channels and segmentation of high values of green and red is done to identify overlap (yellow). The resulting binary image is saved in an output directory. Developed in ImageJ Macro language (.ijm file).

//To be read in ImageJ with Language ImageJ Macro

// Set the input and output directories

inputDir = getDirectory("Choose Composite Directory");

outputDir = getDirectory("Choose Output Directory for the Yellow binary images");

// Process each file in the input directory

fileList = getFileList(inputDir);

prefix_image = ""; //to be filled in case there is a prefix

sufix_image = ""; //to be filled in case there is a sufix

for (i = 0; i < fileList.length; i++) {

if (endsWith(fileList[i], ".tif") || endsWith(fileList[i], ".tiff")) {

// Open the RGB image

open(inputDir + fileList[i]);

/* This code for the well is going to be adjusted according to the name saved for the composite

that differs depending on how the images were saved

*/

shortName = replace(fileList[i], prefix_image , "");

shortName = replace(shortName, sufix_image , "");

// Print the shortName for debugging

print("Short name: " + shortName);

// Split the RGB channels

run("Split Channels");

selectWindow(fileList[i] + " (red)");

run("Duplicate...", "title=red");

selectWindow(fileList[i] + " (green)");

run("Duplicate...", "title=green");

selectWindow(fileList[i] + " (blue)");

run("Duplicate...", "title=blue");

// Close original channel windows

close(fileList[i] + " (red)");

close(fileList[i] + " (green)");

close(fileList[i] + " (blue)");

// Threshold the red channel

selectWindow("red");

setThreshold(128, 255);

run("Convert to Mask");

run("Duplicate...", "title=red_mask");

// Threshold the green channel

selectWindow("green");

setThreshold(128, 255);

run("Convert to Mask");

//run("Fill Holes");

run("Duplicate...", "title=green_mask");

// Invert the blue channel threshold

selectWindow("blue");

setThreshold(0, 127);

run("Convert to Mask");

run("Duplicate...", "title=blue_mask");

// Perform AND operation between red and green masks

imageCalculator("AND create", "red_mask", "green_mask");

selectWindow("Result of red_mask");

run("Duplicate...", "title=yellow_intermediate");

// Perform AND operation with inverted blue mask

imageCalculator("AND create", "yellow_intermediate", "blue_mask");

selectWindow("Result of yellow_intermediate");

// Rename and save the final yellow segmented image

rename("yellow_segmented_" + shortName);

saveAs("Tiff", outputDir + "yellow_segmented_" + shortName + ".tif");

// Close all images

close("*");

}

}

**Supplementary File 4: Quantification of cells positive for phagocytosis**. This script performs batch processing of binary cell images (Suppl. File 1) and binary yellow images (Suppl. File 3). It identifies regions of overlap between possible phagocytosis regions (yellow) and cells. It results in a new binary image and particle analysis, that are saved in the output directory. Designed in ImageJ Macro language (.ijm file).

//To be read in ImageJ with Language ImageJ Macro

// Set the input and output directories

firstDir = getDirectory("Choose Directory of the Cells Binary");

secondDir = getDirectory("Choose Directory of Yellow");

outputDirectory = getDirectory("Choose Output Directory");

secondFileList = getFileList(secondDir);

//yellow_segmented_ is part of the segmentation on RGB, so it will always be present, if more prefix, add on ""

prefix_yellow = "yellow_segmented_" + "";

//the sufix will always be composed of _composite.tif.tif, if something else add on ""

sufix_yellow = "" + "_composite.tif.tif";

//add if needed

prefix_binary_cells = "";

sufix_binary_cells = "";

for (i = 0; i < secondFileList.length; i++) {

if (endsWith(secondFileList[i], ".tif") || endsWith(secondFileList[i], ".tiff")) {

// Extract the image code from the yellow file name

filename = secondFileList[i];

print(filename);

// Remove the prefix "yellow_segmented_"

filename = replace(filename, prefix_yellow , "");

print(filename);

// Remove the suffix "_00d03h00m_composite.tif.tif"

imageCode = replace(filename, sufix_yellow, "");

print("Image Code: " + imageCode);

// Find the corresponding image in the first directory

firstFile = "";

firstFileList = getFileList(firstDir);

for (j = 0; j < firstFileList.length; j++) {

// Remove the sufix and prefix from the first file name for matching with yellow code

firstFileName = replace(replace(replace(firstFileList[j], prefix_binary_cells, ""), sufix_binary_cells , ""), ".tif", "");

if (firstFileName == imageCode) {

firstFile = firstFileList[j];

print("Matching File: " + firstFile);

break;

}

}

// If a matching file is found, proceed with the operations

if (firstFile != "") {

// Log the image combination

print("Processing: " + secondFileList[i] + " with " + firstFile);

// Open the image from the second directory

open(secondDir + secondFileList[i]);

titleSecond = getTitle();

//run("Threshold...");

setAutoThreshold("Default dark");

//setThreshold(255, 255);

run("Convert to Mask");

// Perform particle analysis with min size 20 and create a mask

run("Analyze Particles...", "size=20-800 show=Masks exclude");

maskTitle = getTitle();

run("Invert LUT");

// Open the corresponding image from the first directory

open(firstDir + firstFile);

titleFirst = getTitle();

// Use Image Calculator to multiply the mask with the image from the first directory

imageCalculator("Multiply create", maskTitle, titleFirst);

// Perform particle analysis on the resulting image with all particles

//setAutoThreshold("Default");

run("Analyze Particles...", "exclude summarize");

// Save the image

saveAs("Tiff", outputDirectory + "Multiply_Result_" + imageCode + ".tif");

// Close all images

close("*");

}

}

}

**Supplementary File 5: Determination of DNA-positive area.** This script performs batch processing of fluorescent images to determine DNA-positive regions. It applies threshold segmentation to the images from the input directory and generates binary images. Particle analysis is used to analyse the regions. The results and binary images are saved in an output directory. Designed in ImageJ Macro language (.ijm file).

// Macro to process a batch of images in ImageJ - ImageJ Macro Language

// Set the input - green channel - and output directories - save binary images

inputDir = getDirectory("Choose the Input Directory ");

outputDir = getDirectory("Choose the Output Directory ");

// Get list of files in the input directory

list = getFileList(inputDir);

// Loop through all files in the directory

for (i=0; i<list.length; i++) {

if (endsWith(list[i], ".tif")) {

// Open the image

open(inputDir + list[i]);

// Subtract Background

run("Subtract Background...", "rolling=25");

// Convert to 8-bit

run("8-bit");

// Set Threshold

setThreshold(34, 255);

// Apply the threshold

setOption("BlackBackground", true);

run("Convert to Mask");

// Save the binary image

saveAs("Tiff", outputDir + list[i]);

// Run Particle Analysis

run("Set Measurements...", "area redirect=None decimal=3");

run("Analyze Particles...", "size=0-Infinity display summarize");

// Save the Results

//saveAs("Results", outputDir + replace(list[i], ".tif", ".csv"));

// Close all images and results

close("*");

}

}

**Supplementary File 6:** Alignment of Sanger Sequencing DNA results for PCR2 with CFTR mRNA. PCR2 products for the 3 biological replicas are annotated as follows: sequences 1-2, 3-4 and 5-6 are from biological replicas numbers 2, 3 and 1, respectively. Sequences 1, 3 and 5 were generated starting from the Forward Primer. Sequences 2, 4 and 6 were generated starting the Reverse Primer. CFTR mRNA with Consensus CDS CCDS5773.1. Alignment generated with SnapGene.


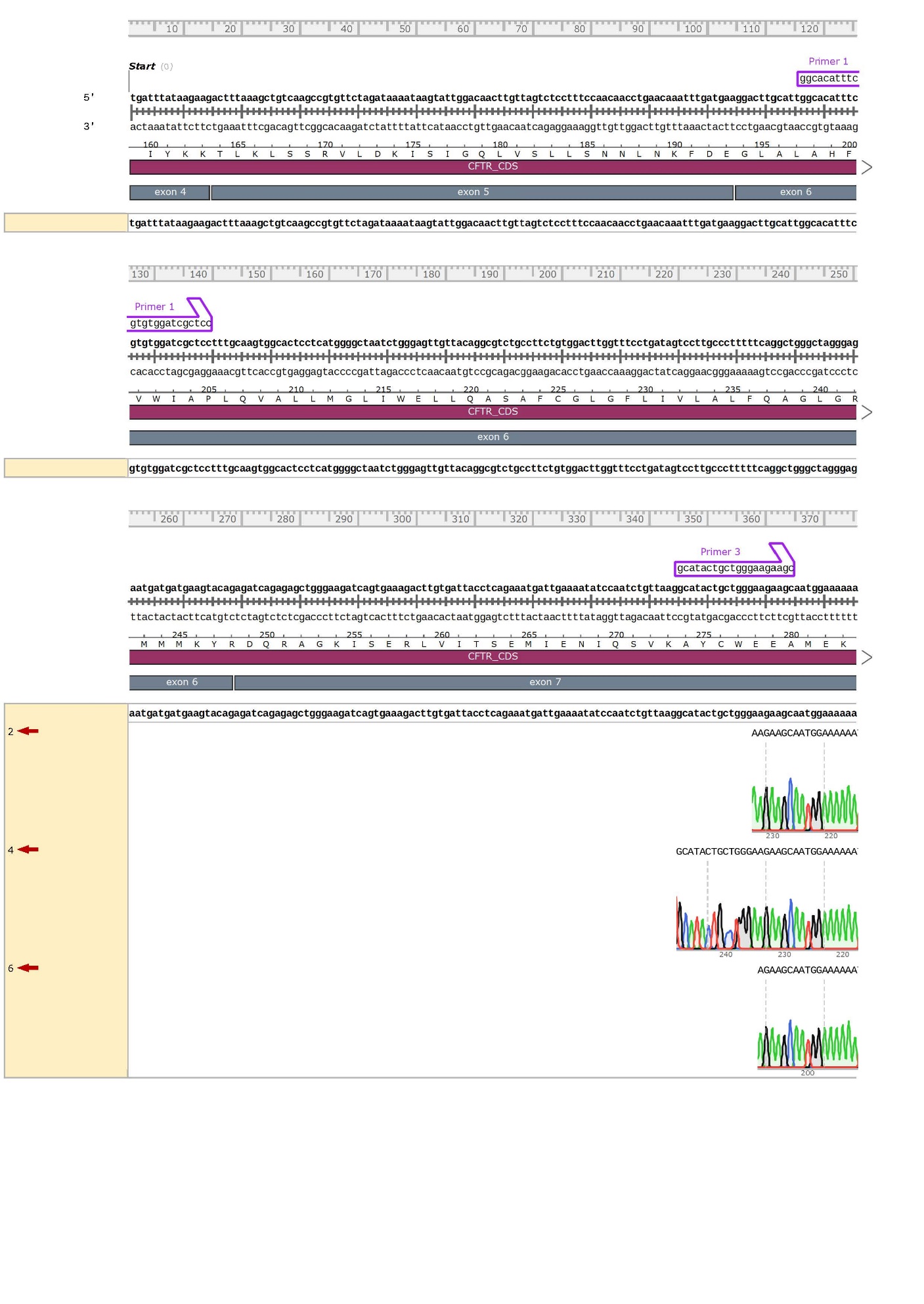


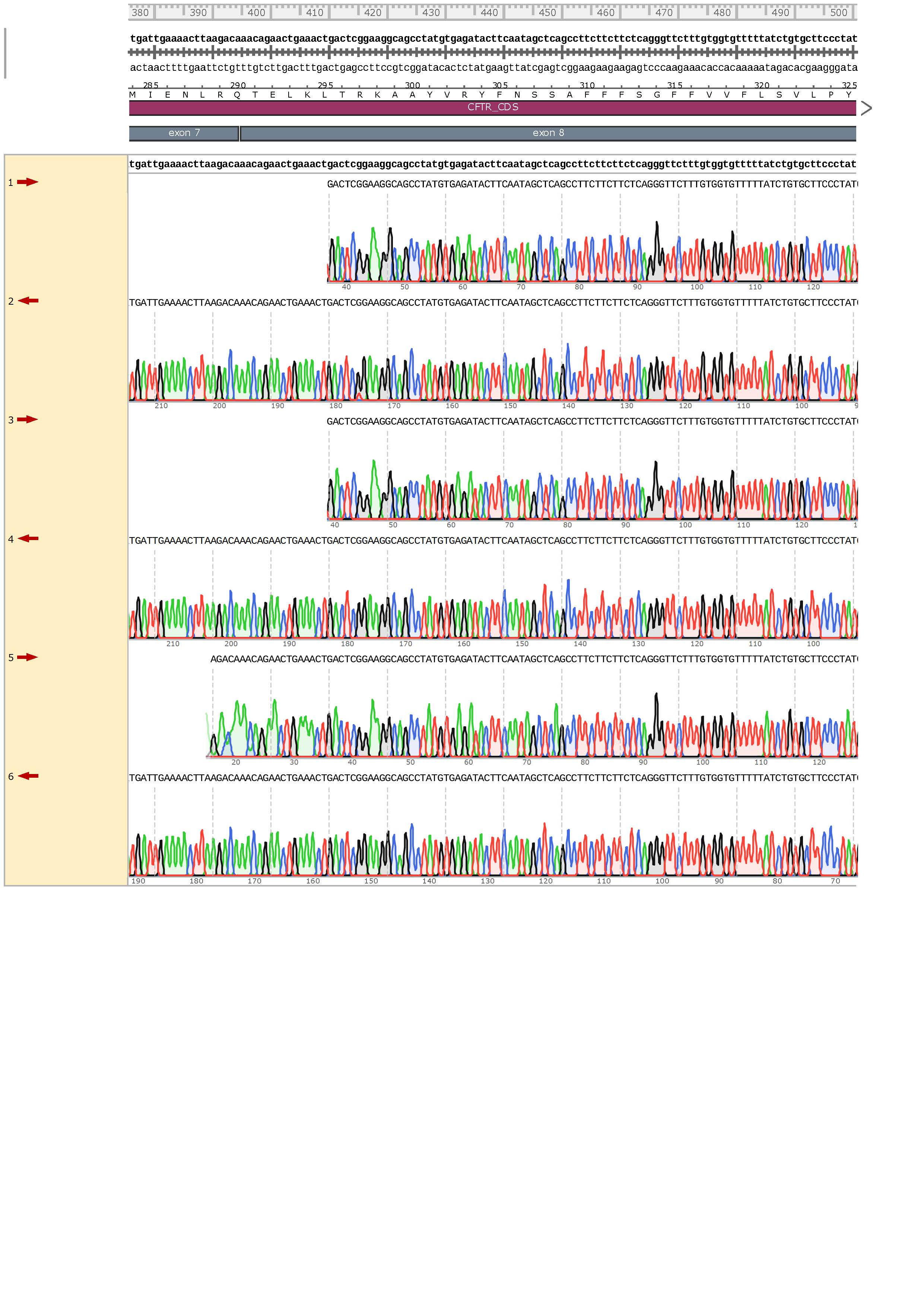


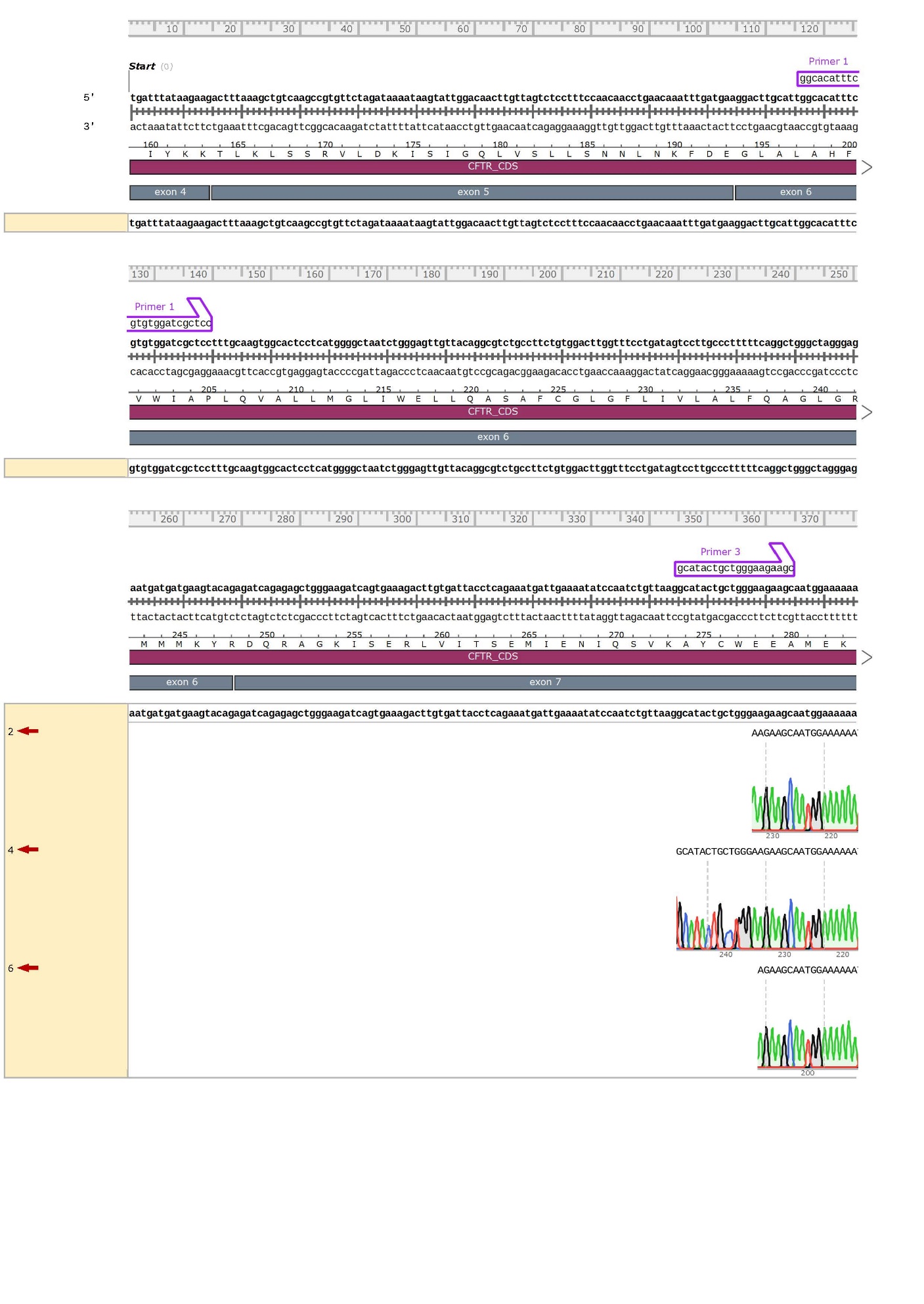

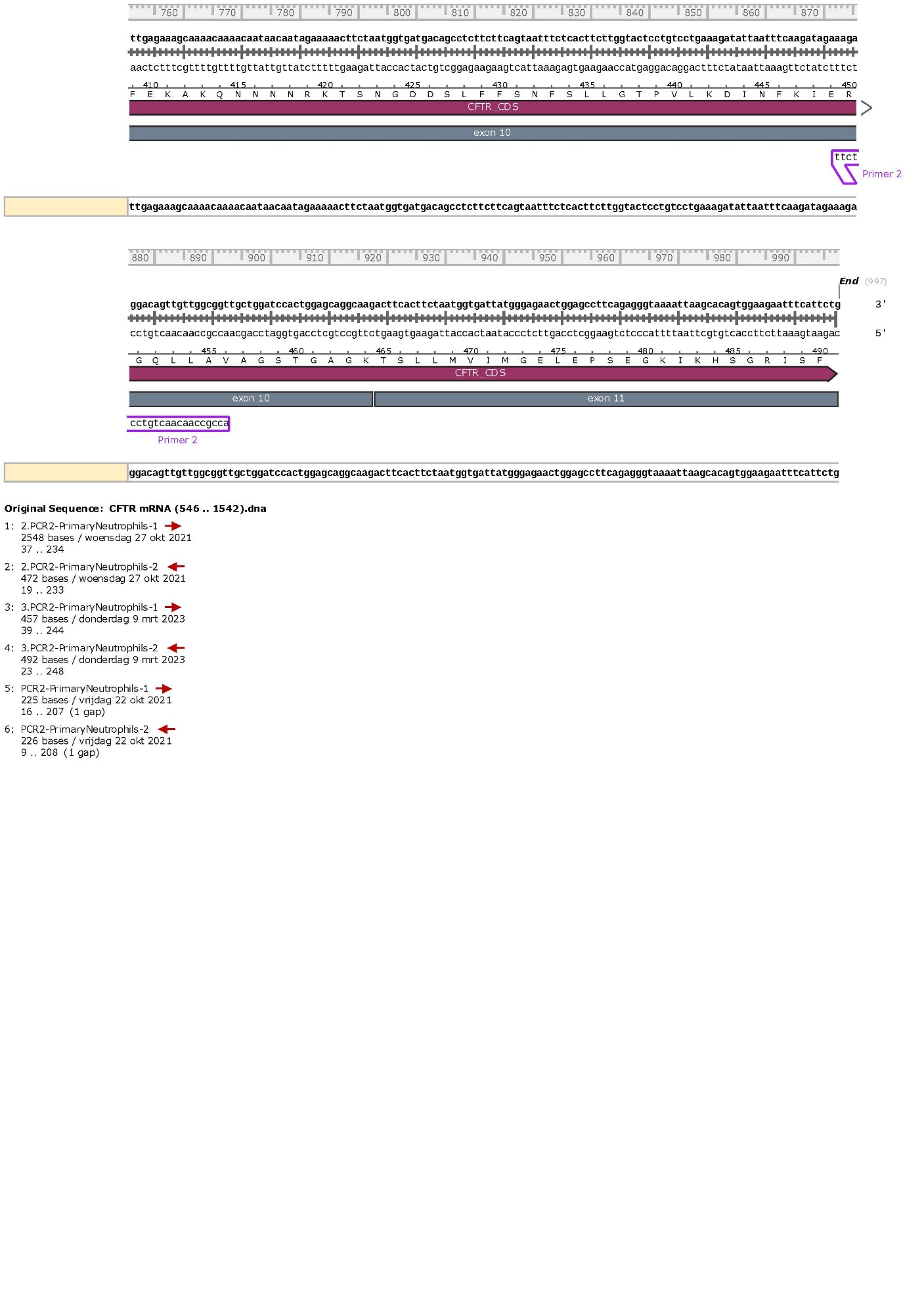


**Table S1:** Table with all catalog numbers (alphabetical).

| **Reagent** | **Company** | **Catalog number** |
| --- | --- | --- |
| AF555-Phalloidin | Invitrogen | A34055 |
| BD CytoFix/CytoPerm | BD Biosciences | 554714 |
| BD Perm/Wash | BD Biosciences | 554714 |
| Bovine serum albumin 7.5% | Gibco | 15260-037 |
| BSA | Sigma-Aldrich | A7906-50G |
| C5A | R&D Systems | 2037-C5-025 |
| DMSO | Sigma-Aldrich | D2650 |
| Donkey Anti-Mouse IgG H&L (Alexa Fluor® 488) (ab150105) | Abcam | ab150105 |
| D-PBS | VWR | 392-0434 |
| DTT | Alfa Aesar GmbH | J15397-06 |
| EasyEights magnet | StemCell Technologies | 18103 |
| EasySep Direct Human Neutrophil Isolation kit | StemCell Technologies | 19666 |
| EDTA | Sigma-Aldrich | E5134 |
| EDTA-coated tube | BD Biosciences | 367525 |
| FBS, qualified, USDA-approved regions (reserve number 1802332) | Life Technologies | 10437028 |
| FCS | Sigma-Aldrich | F7524 |
| Fibronectin from human plasma | Thermo Fisher | 33016-015 |
| fMLP | Sigma-Aldrich | F3506 |
| Genelute PCR Clean-up Kit | Sigma-Aldrich | NA1020 |
| Gentamicin | Thermo Fisher | 15750045 |
| GM-CSF | Peprotech | 300-03 |
| HBSS buffer | Gibco | 14065-049 |
| High-Capacity cDNA Reverse Transcription Kit | Applied Biosystems™ | 4368814 |
| HSA | Belgian Red Cross | / |
| Human Bronchial Epithelial Cell Line 16HBE14o- | Sigma-Aldrich | SCC150 |
| IL-8 | Peprotech | 200-08M |
| Inh-172 | Selleckchem (Bio-Connect) | S7139 |
| LHC-8 basal medium | Gibco | 12677-027 |
| LPS | Sigma-Aldrich | L2630 |
| LTB4 | Cayman chemical | 20110 |
| Luminol | Sigma-Aldrich | 123072 |
| MacConkey agar plates | Sigma-Aldrich | 70143 |
| MEM | Gibco | 11095-072 |
| Mouse monoclonal anti-human CFTR antibodies (CFTR antibody mix 570, 596 & 660) | CFFT labs | / |
| MPO ELISA | R&D Systems | DY3174 |
| NE ELISA | R&D Systems | DY9167-05 |
| Nunc™ Lab-Tek™ Chambered Coverglass | Thermo Scientific™ | 155411 |
| Paraformaldehyde | MP Biomedicals | 199983 |
| PGN | Sigma-Aldrich | 77140 |
| pHrodo-beads S. aureus | Invitrogen | A10010 |
| PMA | Sigma-Aldrich | P8139 |
| Poly-L-lysine | Sigma-Aldrich | P6282 |
| PureCol | Merck | 5006-15MG |
| Rneasy® Micro Kit | Qiagen | 74004 |
| RPMI medium | Gibco | 32404-014 |
| Saponin | Sigma-Aldrich | S4521 |
| Sytox Green | Invitrogen | S7020 |
| Taq DNA Polymerase, recombinant (1 U/µL) | Fisher Scientific | EP0404 |
| Triton-X | Alfa Aesar | A16046 |
| Trypan Blue stain 0,4% | Thermo Fisher | 15250061 |
| TrypLE™ Express Enzyme (1X), phenol red | Thermo Fisher | 12605010 |
| Türk’s solution | Sigma-Aldrich | 1.09277.0100 |
| ViewPlate-96, White 96-well Microplate with Clear Bottom, Sterile and Tissue Culture Treated, Lid Included | PerkinElmer | 6005181 |
